# Residential mobility restrictions and adverse mental health outcomes during the COVID-19 pandemic in the UK

**DOI:** 10.1038/s41598-024-51854-6

**Published:** 2024-01-20

**Authors:** Ho Fai Chan, Zhiming Cheng, Silvia Mendolia, Alfredo R. Paloyo, Massimiliano Tani, Damon Proulx, David A. Savage, Benno Torgler

**Affiliations:** 1 School of Economics and Finance, Queensland University of Technology, Brisbane, QLD 4000 Australia; 2 Centre for Behavioural Economics, Society and Technology (BEST), Brisbane, QLD 4000 Australia; 3 Centre for Behavioural Insights for Technology Adoption (BITA), Brisbane, QLD 4000 Australia; 4 Social Policy Research Centre, University of New South Wales, Kensington, NSW 2052 Australia; 5 Department of Management, Macquarie Business School, Macquarie University, Sydney, NSW 2109 Australia; 6 Department of Economics, Social Studies and Applied Mathematics and Statistics, University of Turin, Turin, Italy; 7 University of Wollongong, Keiraville, NSW 2500 Australia; 8 School of Business, UNSW Canberra, Canberra, ACT Australia; 9 Newcastle Business School, University of Newcastle, Newcastle, NSW Australia; 10 CREMA – Center for Research in Economics, Management and the Arts, Basel, Switzerland

**Keywords:** Diseases, Health care

## Abstract

During the COVID-19 pandemic, several governments tried to contain the spread of SARS-CoV-2, the virus that causes COVID-19, with lockdowns that prohibited leaving one’s residence unless carrying out a few essential services. We investigate the relationship between limitations to mobility and mental health in the UK during the first year and a half of the pandemic using a unique combination of high-frequency mobility data from Google and monthly longitudinal data collected through the Understanding Society survey. We find a strong and statistically robust correlation between mobility data and mental health survey data and show that increased residential stationarity is associated with the deterioration of mental wellbeing even when regional COVID-19 prevalence and lockdown stringency are controlled for. The relationship is heterogeneous, as higher levels of distress are seen in young, healthy people living alone; and in women, especially if they have young children.

## Introduction

Daily mobility is crucial for maintaining good mental health and overall wellbeing. Research has shown that individuals whose daily activities are limited to their immediate residential neighbourhood are at a higher risk of developing depression than those with a wider activity space beyond their immediate locality^1^. Time and space inequalities resulting from fears of environmental factors such as crime also negatively affect mental health, as they can reduce individuals’ ability to access and utilise different spaces and times within their immediate and broader environment^2^.

The COVID-19 pandemic profoundly impacted daily mobility and social connections. Governments’ recourse to lockdowns—the prohibition against leaving one’s residence unless carrying out a few essential services—to contain the spread of COVID-19 has compounded their adverse effects on people’s mental health status^3–6^.

However, while there is little doubt that the pandemic has had a heavy toll on households’ stress levels, the verdict on lockdowns as an effective nonpharmaceutical intervention to slow or prevent the spread of disease is less clear-cut. On the one hand, lockdowns have been effective in several settings, both in ex-ante modelling exercises^7^ and ex-post evaluations^8–10^. On the other hand, by reducing the spread of COVID-19 and the transmission of SARS-CoV-2, lockdowns likely improved the mental wellbeing of the population by reducing the fear of being infected^11^, the possibility of family and friends dying of COVID-19, and the financial insecurity brought about by pandemic-related reduced economic activity^12^. A priori, it is not clear which impact on mental health—the negative one associated with physical isolation or the positive one associated with reduced prevalence of COVID-19—is larger in magnitude, nor is it clear if the net effect switches from benefiting to harming society when restrictions last beyond a certain point.

Our objective is to answer the following research question: what is the association between a local measure of mobility and wellbeing in the UK during the COVID-19 pandemic? We focus on the relationship between restrictions to mobility and individuals’ mental wellbeing in the UK, adding to both literatures examining mental wellbeing during the pandemic^13–19^ and specific studies for the United Kingdom^5,20–35^.

Our study makes a methodological contribution by combining monthly data on wellbeing collected at the local authority district’s level by Understanding Society—a longitudinal survey of representative households across the UK—with high-frequency daily data on levels of mobility collected by Google’s publicly available Community Mobility Reports in the same areas. We study spatial variation in the 12-item General Health Questionnaire (GHQ-12) “Caseness score”^36^, which refers to the presence of symptoms that meet the diagnostic criteria for depression and anxiety to be classed as a clinical case, with variation in the time spent at home obtained through Google’s Community Mobility Reports. The use of this variable improves on studies that rely on an event-study approach (e.g.,^5^) using as triggers the dates in which lockdowns took effect. To disentangle the specific influence of restrictions in mobility on mental wellbeing we apply individual, regional, and wave-fixed effects. We also include detailed information on participants’ pre-pandemic traits.

In summary, as a preview of our results, we find that a one standard deviation increase in residential mobility (which equals about 6 pp difference compared to pre-pandemic level) is associated with a 0.066–0.084 standard deviations increase in the GHQ Caseness score [95% CI 0.021–0.112]. There is some evidence of heterogeneity in the extent to which mental wellbeing can be affected by the amount of time spent at home. It appears that older people, men, and those with partners exhibit weaker correlations, as well as those people living in rural areas, those who own their houses outright (i.e., not paying off a mortgage), those who were not working, and those who were less healthy.

## Materials and methods

### Understanding society (UK Household Longitudinal Study)

We use data from the UK Household Longitudinal Study (UKHLS), now known as Understanding Society^37^*.* UKHLS surveyed approximately 40,000 households living in the United Kingdom in Wave 1. The survey contains a wide range of questions on social, economic, and behavioural issues. Respondents lived in 380 Local Authority Districts (LAD) across the UK. Data collection started in 2009–2010 for Wave 1; ten waves are currently available. Wave 10 (pre-COVID-19) consists of individuals surveyed during the period 2018–2019.

On April 2020, selected respondents of the Understanding Society were invited to take part in the first wave of a new COVID-19 special survey, which consisted of important questions on the impact of the pandemic on the wellbeing of individuals, families, and wider communities, including information about caring responsibilities and family life, employment and financial situation, financial wellbeing, homeschooling, and mental wellbeing. Participants were asked to complete one survey per month until July 2020, followed by a survey every two months from September 2020 to track changes in their circumstances and environments. 17,452 individuals completed a full post-COVID-19 survey in April 2020^38^. Approval for all data collection related to the Understanding Society main study and innovation panel waves has been granted by the Ethics Committee of the University of Essex (see https://www.understandingsociety.ac.uk/documentation/mainstage/user-guides/main-survey-user-guide/ethics). Consent to participating in the survey was obtained from all subjects. Supplementary Table 1 presents basic descriptive statistics of the estimation sample.

Our analysis is based on Wave 10 of the regular Understanding Society and the nine waves (April, May, June, July, September, and November 2020, and January, March, and September 2021) of the Understanding Society COVID-19 special survey. The final estimation sample consists of 110,008 (individual × wave) observations, with 19,763 individuals from 13,295 households. Thus, the analysis below covers the period of the initial lockdown and a series of repeated lockdowns until one and a half years into the pandemic—from March 2020 to September 2021.

Mental health was measured in Understanding Society using the General Health Questionnaire (GHQ) Caseness score^39,40^. The GHQ is regarded as one of the most reliable indicators of psychological distress or “disutility”^41,42^, see Supplementary Note 1 for question wordings and has been used in previous literature in various fields (in studies from psychology^43^ and other social sciences^44–47^, among others). Previous studies^6,48^ have proxied mental wellbeing using Google Trends data generated by searches performed on Google. Another stream of studies has used helpline calls to examine the impact of COVID-19 on mental health^49–52^.

The GHQ Caseness score is constructed from responses to 12 questions covering feelings of strain, depression, inability to cope, anxiety-based insomnia and lack of confidence (see the Supplementary information for details). The twelve answers are summed up into a GHQ Caseness score that indicates the level of mental distress, resulting in a scale from 0 (the least distressed) to 12 (the most distressed).

### Google’s community mobility reports

The impact of the UK’s series of lockdowns on mobility is measured using movement information collected by Google from internet-connected devices with the “location history” setting turned on. The information is anonymised, and a person cannot be identified from the resulting datasets called Community Mobility Reports^53,54^. These reports are provided by Google to the public, partly to assist in crafting policies that can help limit the spread of SARS-CoV-2. For our analysis, we focus on the mobility measure relating to time spent in residential places, i.e., changes in the length of stay at home compared to the pre-COVID-19 baseline, which is the median value of the corresponding day of the week between the 3rd January and 6th February 2020. We use a restricted version of Understanding Society including information on the Local Authority District where respondents live at each wave and we link this with Google Mobility Reports’ measures on the same refined geographical level (i.e., nomenclature of territorial units for statistics (NUTS) level 3). We show the average monthly changes in residential mobility across the 12 regions in the UK in Supplementary Fig. 1. We observe that London dwellers had the largest decrease in mobility at the beginning of the pandemic (i.e., April 2020), as the duration of staying home increased by approximately 30% compared to the January 2020 baseline. For subsequent analysis, we calculate, for each participant at the LAD level, the average mobility change in the past 7 (or 14) days from the date when the survey was conducted.

We employ data from Google’s “Community Mobility Reports”, acknowledging upfront that the dataset is generated from individuals who own internet-connected devices with the “location history” setting activated. This may potentially introduce a bias towards specific demographic or socioeconomic groups who are more likely to have such devices and use these settings.

However, the use of these datasets offers significant advantages, mainly due to their high volume and high velocity. Despite potential selection bias, the sheer scale of the data encompasses a substantial proportion of the population, often providing a valuable approximation of broad trends and patterns.

The large volume of data points in Google’s “Community Mobility Reports” allows for a granular analysis. Geographic or demographic stratification is possible, which can identify trends and insights that might not be evident with smaller datasets. Moreover, the real-time or near real-time nature of these datasets provides almost instantaneous feedback on changes in mobility patterns. This is a particularly crucial aspect in rapidly evolving situations such as pandemics, natural disasters, or major societal events.

Recognizing both the potential bias and the unique opportunities provided by these datasets, we believe they offer a powerful tool for understanding community mobility. They provide insights on a scale and at a velocity that would be challenging, if not impossible, to obtain using traditional data sources. As we proceed with our analysis, we keep these considerations at the forefront, interpreting our findings within the context of the inherent strengths and limitations of these high-volume and high-velocity datasets.

### COVID statistics and government stringency

We use data on cases and death due to COVID-19 obtained from the UK Health Security Agency, which was reported up to the level of the 12 Government Office Regions: North East, North West, Yorkshire and the Humber, East Midlands, West Midlands, East of England, London, South East, South West, Wales, Scotland, and Northern Ireland. This information is combined with the population to calculate the reported case per thousand people at the regional level. Thus, in our study, we are also able to control for the severity of COVID-19 in the region, which allows us to account for the changes in mental health associated with the prevalence of COVID-19 within a region, similar to how Brodeur et al.^6^ used lagged COVID-19-related deaths.

We use the COVID-19 Stringency Index from the Oxford Coronavirus Government Response Tracker (OxCGRT) to proxy the strictness of lockdown policies implemented by the government^55^. The stringency index is a composite measure constructed from nine policy indicators (e.g., workplace closures; restrictions on public gatherings; closures of public transport; stay-at-home requirements) and is reported at the UK country levels (England, Scotland, Wales, and Northern Ireland, see Supplementary Fig. 2 for the development of Stringency Index over February 2020 to September 2021 across the four countries). Like the mobility measure, we calculate the past 7-day (or 14-day) average of COVID-19 case statistics and stringency index from the date the survey was conducted.

Summary statistics are presented in Supplementary Table 1.

### Estimation strategy

We model individual mental health—measured using the GHQ Caseness score—as a function of the change in the duration spent at home, socio-economic and demographic characteristics, mental health stock before the pandemic, COVID-19 prevalence in the community, time-invariant region and individual fixed effects, as well as period fixed effects. More explicitly, we estimate variations of the following regression equation:

$$GHQ_{it} = \alpha + \beta \left( {\Delta {\text{home}}} \right)_{rt} + {\mathbf{\gamma^{\prime}X}}_{it} + \varepsilon_{it} ,$$

where $i$ and $t$ are individual and wave indexes, respectively, $\left( {\Delta {\text{home}}} \right)_{rt}$ is the past 7-day average of the percentage change in time spent at home compared to the pre-pandemic baseline period in area *r* at the time of survey *t*, ${\mathbf{X}}_{it}$ is a vector of control variables as described previously, and $\varepsilon_{it}$ is the error term. The parameters $\alpha$, $\beta$, and vector of parameters ${{\varvec{\upgamma}}}$ are estimated via generalised least squares. The standard errors presented in Tables 1, 2 and 3 are robust to autocorrelation and heteroskedasticity; clustering at the region-by-wave level does not change our substantive results. The parameter of interest is $\beta$, which quantifies the relationship between the GHQ Caseness score and the change in the duration of time spent at home.

**Table 1:** Coefficient estimates of the relationship between mental wellbeing and residential mobility.

	(1)	(2)	(3)	(4)	(5)	(6)	(7)	(8)
Increase in duration of time spent at home (%)	0.043***	0.044***	0.044***	0.042***	0.042***	0.025***	0.025**	0.024**
	(0.00126)	(0.00130)	(0.00130)	(0.00185)	(0.00188)	(0.00656)	(0.00839)	(0.00827)
COVID-19 positive		0.22***	0.20***	0.17***	0.17***	0.14***	0.14***	0.12**
		(0.0345)	(0.0339)	(0.0351)	(0.0352)	(0.0363)	(0.0365)	(0.0404)
Age		− 0.018***	− 0.014***	− 0.015***	− 0.015***	− 0.015***	− 0.015***	
		(0.00203)	(0.00187)	(0.00192)	(0.00192)	(0.00192)	(0.00194)	
Female		0.75***	0.56***	0.55***	0.55***	0.55***	0.55***	
		(0.0407)	(0.0368)	(0.0375)	(0.0375)	(0.0375)	(0.0372)	
*Marital status*								
Married/civil partnership		− 0.31***	− 0.19**	− 0.17**	− 0.16*	− 0.16*	− 0.15*	
		(0.0681)	(0.0623)	(0.0635)	(0.0635)	(0.0635)	(0.0640)	
Separated/divorced/widowed		0.015	− 0.0082	0.0069	0.0035	0.0047	0.0089	
		(0.0849)	(0.0752)	(0.0765)	(0.0766)	(0.0766)	(0.0771)	
Living with partner		− 0.29***	− 0.21***	− 0.23***	− 0.23***	− 0.23***	− 0.23***	− 0.17**
		(0.0459)	(0.0442)	(0.0455)	(0.0455)	(0.0455)	(0.0458)	(0.0581)
*Education*		− 0.24*	− 0.31**	− 0.29**	− 0.29**	− 0.29**	− 0.28**	
No qualification		(0.115)	(0.0986)	(0.101)	(0.101)	(0.101)	(0.103)	
		− 0.12	− 0.14^†^	− 0.16*	− 0.16^†^	− 0.16^†^	− 0.17*	
Other qualification		(0.0927)	(0.0803)	(0.0810)	(0.0811)	(0.0811)	(0.0812)	
		− 0.015	0.0048	0.014	0.011	0.011	0.012	
A level		(0.0670)	(0.0598)	(0.0612)	(0.0612)	(0.0612)	(0.0612)	
		0.062	0.083	0.088	0.084	0.088	0.086	
Other higher degree		(0.0736)	(0.0654)	(0.0667)	(0.0667)	(0.0668)	(0.0666)	
		0.16**	0.16**	0.16**	0.17**	0.18**	0.17**	
Degree		(0.0598)	(0.0537)	(0.0548)	(0.0552)	(0.0553)	(0.0560)	
		− 0.057	− 0.029	− 0.0090	− 0.0059	− 0.025	− 0.000032	
Live in rural area		(0.0454)	(0.0408)	(0.0419)	(0.0433)	(0.0435)	(0.0523)	
*Housing status*								
Mortgage		0.28***	0.13**	0.12*	0.12*	0.12*	0.12*	
		(0.0532)	(0.0482)	(0.0493)	(0.0493)	(0.0493)	(0.0501)	
Renting		0.77***	0.34***	0.33***	0.34***	0.34***	0.32***	
		(0.0701)	(0.0620)	(0.0632)	(0.0635)	(0.0635)	(0.0636)	
*Employment*								
Unemployed		0.54***	0.38***	0.38***	0.38***	0.38***	0.39***	0.46***
		(0.0475)	(0.0452)	(0.0471)	(0.0470)	(0.0471)	(0.0473)	(0.0710)
Self-employed		0.22***	0.22***	0.23***	0.23***	0.23***	0.22***	0.091
		(0.0612)	(0.0576)	(0.0581)	(0.0581)	(0.0581)	(0.0586)	(0.0975)
*Household composition*								
Aged 0–4		0.024	0.075	0.063	0.063	0.062	0.059	
		(0.0514)	(0.0495)	(0.0506)	(0.0505)	(0.0505)	(0.0507)	
Aged 5–15		0.059*	0.058*	0.067*	0.066*	0.067*	0.066*	
		(0.0291)	(0.0273)	(0.0281)	(0.0281)	(0.0281)	(0.0283)	
Aged 70 or older		− 0.099*	− 0.068^†^	− 0.052	− 0.051	− 0.052	− 0.050	
		(0.0436)	(0.0415)	(0.0427)	(0.0427)	(0.0427)	(0.0428)	
Pre-COVID GHQ (Caseness score)			0.38***	0.38***	0.38***	0.38***	0.38***	
			(0.00829)	(0.00845)	(0.00845)	(0.00845)	(0.00837)	
Long-standing illness or impairment			0.49***	0.51***	0.51***	0.51***	0.50***	
			(0.0415)	(0.0423)	(0.0423)	(0.0423)	(0.0422)	
Case per 1,000 people				3.87***	3.74***	0.88	0.71	1.09
				(0.720)	(0.735)	(0.969)	(1.001)	(0.996)
Stringency index				0.0018**	0.0017**	0.0049^†^	0.0050^†^	0.0056*
				(0.000582)	(0.000589)	(0.00275)	(0.00278)	(0.00275)
Constant	1.75***	2.04***	1.19***	1.09***	1.04***	1.22***	0.99*	1.57***
	(0.0276)	(0.126)	(0.116)	(0.120)	(0.134)	(0.277)	(0.481)	(0.270)
Region FE	No	No	No	No	Yes	Yes	No	Yes
LAD FE	No	No	No	No	No	No	Yes	No
Wave FE	No	No	No	No	No	Yes	Yes	Yes
Individual FE	No	No	No	No	No	No	Yes	Yes
Observations	116,513	110,840	109,268	101,236	101,236	101,236	101,236	108,001
Individuals (cluster)	18,517	17,361	17,049	16,662	16,662	16,662	16,662	18,073
R^2^-within	0.015	0.016	0.016	0.017	0.017	0.019	0.019	0.019
R^2^-between	0.004	0.078	0.259	0.261	0.262	0.262	0.279	0.008
R^2^-overall	0.007	0.057	0.183	0.183	0.183	0.184	0.196	0.009
Prob. > *F*	0.000	0.000	0.000	0.000	0.000	0.000	0.000	0.000

Generalised least squares regressions. Dependent variable: Mental wellbeing (GHQ Caseness score). Reference group: *Male, Single, Not living with a partner, GCSE, Living in urban area, Owned a house outright,* and *Employed.* Standard errors are robust to autocorrelation and heteroskedasticity. †*p* < 0.10; *p < 0.05; **p < 0.01; ***p < 0.001.

**Table 2:** Heterogeneity over age and indicators for gender, marital status, living with a partner, and educational attainment.

Subjective wellbeing (GHQ): Caseness score	(1)	(2)	(3)	(4)	(5)
	Age	Gender	Marital status	Living with partner	Education
Increase in duration of time spent at home (%) (Δ	0.058***	0.011^†^	0.030***	0.027***	0.022**
home duration)	(0.00824)	(0.00669)	(0.00719)	(0.00701)	(0.00719)
Age*Δ home duration	− 0.00062***				
	(0.0000860)				
Female*Δ home duration		0.023***			
		(0.00263)			
Married/civil partnership*Δ home duration			− 0.0073*		
			(0.00356)		
Separated/divorced/widowed*Δ home duration			− 0.0051		
			(0.00475)		
Living with partner*Δ home duration				− 0.0035	
				(0.00313)	
No qualification*Δ home duration					− 0.012^†^
					(0.00671)
Other qualification*Δ home duration					− 0.010^†^
					(0.00582)
A level*Δ home duration					0.0046
					(0.00441)
Other higher degree*Δ home duration					0.00023
					(0.00475)
Degree*Δ home duration					0.0047
					(0.00387)
Age	− 0.0056*	− 0.015***	− 0.015***	− 0.015***	− 0.015***
	(0.00227)	(0.00192)	(0.00192)	(0.00192)	(0.00192)
Female	0.55***	0.22***	0.55***	0.55***	0.55***
	(0.0375)	(0.0525)	(0.0375)	(0.0375)	(0.0375)
*Marital status*					
Married/civil partnership	− 0.16**	− 0.16*	− 0.055	− 0.16*	− 0.16*
	(0.0635)	(0.0635)	(0.0812)	(0.0635)	(0.0635)
Separated/divorced/widowed	0.0072	0.0058	0.080	0.0048	0.0054
	(0.0767)	(0.0766)	(0.103)	(0.0766)	(0.0766)
Living with partner	− 0.22***	− 0.23***	− 0.23***	− 0.18**	− 0.23***
	(0.0455)	(0.0455)	(0.0455)	(0.0630)	(0.0455)
*Education*					
No qualification	− 0.29**	− 0.29**	− 0.29**	− 0.29**	− 0.13
	(0.101)	(0.101)	(0.101)	(0.101)	(0.138)
Other qualification	− 0.16^†^	− 0.15^†^	− 0.15^†^	− 0.15^†^	− 0.0044
	(0.0811)	(0.0811)	(0.0811)	(0.0811)	(0.116)
A level	0.012	0.011	0.012	0.011	− 0.054
	(0.0612)	(0.0612)	(0.0612)	(0.0612)	(0.0861)
Other higher degree	0.089	0.088	0.088	0.088	0.085
	(0.0668)	(0.0668)	(0.0667)	(0.0668)	(0.0926)
Degree	0.18**	0.18**	0.18**	0.18**	0.11
	(0.0553)	(0.0553)	(0.0553)	(0.0553)	(0.0771)
Constant	0.70*	1.43***	1.14***	1.19***	1.26***
	(0.286)	(0.277)	(0.279)	(0.279)	(0.279)
Other Control variables	Yes	Yes	Yes	Yes	Yes
Region fixed-effects	Yes	Yes	Yes	Yes	Yes
Wave fixed-effects	Yes	Yes	Yes	Yes	Yes
N	101,236	101,236	101,236	101,236	101,236
N (cluster)	16,662	16,662	16,662	16,662	16,662
R^2^-within	0.019	0.020	0.019	0.019	0.019
R^2^-between	0.262	0.262	0.262	0.262	0.262
R^2^-overall	0.185	0.185	0.184	0.184	0.184
Prob. > *F*	0.000	0.000	0.000	0.000	0.000

Other control variables include: having ever been COVID positive, living in rural areas, housing status, employment, household composition, pre-COVID mental health, long-term illness, COVID case statistics, and stringency index. Reference group: *Male, Single, Not living with a partner,* and *GCSE.* Standard errors are robust to autocorrelation and heteroskedasticity. †*p* < 0.10; *p < 0.05; **p < 0.01; ***p < 0.001.

**Table 3:** Heterogeneity over indicators for urbanity, home ownership, employment status, pre-existing mental health, and long-term illness.

Subjective wellbeing (GHQ): Caseness score	(6)	(7)	(8)	(9)	(10)
	Live in rural area	Home ownership	Employment status	Pre-existing mental health	Long-term illness
Increase in duration of time spent at home	0.027***	0.019**	0.028***	0.027***	0.029***
(%) (Δ home duration)	(0.00663)	(0.00667)	(0.00672)	(0.00660)	(0.00664)
Live in rural area*Δ home duration	− 0.0083**				
	(0.00307)				
Mortgage*Δ home duration		0.013***			
		(0.00289)			
Renting*Δ home duration		0.0014			
		(0.00410)			
Unemployed*Δ home duration			− 0.0096***		
			(0.00282)		
Self-employed*Δ home duration			0.0031		
			(0.00482)		
Pre-COVID GHQ (Caseness score)*Δ home duration				− 0.0011^†^	
				(0.000578)	
Long-standing illness*Δ home duration					− 0.014***
					(0.00290)
Live in rural area	0.089	− 0.026	− 0.025	− 0.024	− 0.025
	(0.0591)	(0.0435)	(0.0435)	(0.0435)	(0.0435)
Mortgage	0.12*	− 0.076	0.12*	0.12*	0.12*
	(0.0493)	(0.0632)	(0.0493)	(0.0493)	(0.0493)
Renting	0.34***	0.32***	0.34***	0.34***	0.34***
	(0.0635)	(0.0880)	(0.0635)	(0.0635)	(0.0635)
Unemployed	0.38***	0.38***	0.52***	0.38***	0.38***
	(0.0471)	(0.0471)	(0.0608)	(0.0471)	(0.0471)
Self-employed	0.23***	0.23***	0.18*	0.23***	0.23***
	(0.0582)	(0.0582)	(0.0900)	(0.0581)	(0.0582)
Pre-COVID GHQ (Caseness)	0.38***	0.38***	0.38***	0.40***	0.38***
	(0.00845)	(0.00845)	(0.00845)	(0.0122)	(0.00845)
Long-standing illness or impairment	0.51***	0.51***	0.51***	0.51***	0.71***
	(0.0423)	(0.0423)	(0.0423)	(0.0423)	(0.0588)
Constant	1.20***	1.30***	1.18***	1.20***	1.16***
	(0.277)	(0.277)	(0.277)	(0.277)	(0.277)
Other control variables	Yes	Yes	Yes	Yes	Yes
Region fixed-effects	Yes	Yes	Yes	Yes	Yes
Wave fixed-effects	Yes	Yes	Yes	Yes	Yes
N	101,236	101,236	101,236	101,236	101,236
N (cluster)	16,662	16,662	16,662	16,662	16,662
R^2^-within	0.019	0.019	0.019	0.019	0.019
R^2^-between	0.262	0.262	0.262	0.262	0.263
R^2^-overall	0.184	0.184	0.184	0.184	0.184
Prob. > *F*	0.000	0.000	0.000	0.000	0.000

Other control variables include: having ever been COVID positive, age, gender, marital status, living with a partner, education, household composition, COVID case statistics, and stringency index*.* Standard errors are robust to autocorrelation and heteroskedasticity. †*p* < 0.10; *p < 0.05; **p < 0.01; ***p < 0.001.

To demonstrate the stability of the estimated coefficient, we progressively expand the set of control variables entering ${\mathbf{X}}_{it}$. After the bivariate regression between GHQ and change in home time, we first include a set of socio-economic and demographic characteristics, such as gender, marital status, educational attainment, household composition (having children of various ages), homeownership status (or renting), urban area, and employment status. Information on marital status, educational attainment, urban area, and homeownership status are from the pre-COVID-19 wave. Second, we include a measure of mental health stock using pre-COVID-19 GHQ Caseness score as a proxy, and we control for whether the individual has a long-standing (or chronic) illness. Third, we incorporate the prevalence of COVID-19 in the community by including the number of cases per 1,000 people as well as an index of the stringency of the lockdown. Finally, we include various fixed effects in the regression: individual-specific, region-specific, LAD-specific, and wave-specific fixed effects. These are intended to capture unspecific features or time-unvarying characteristics of individuals, regions, and the survey waves. We explore the heterogeneity of the relationship by interacting the change in home duration with the control variables in a moderator-type analysis.

We have also estimated a version of the model where the GHQ score was coded as a binary measure of psychological distress (using a threshold of 3 or more, which is commonly used as an indicator of mental distress). Results are not included for parsimony but are very similar to those presented in the paper.

## Results

**Figure 1:**
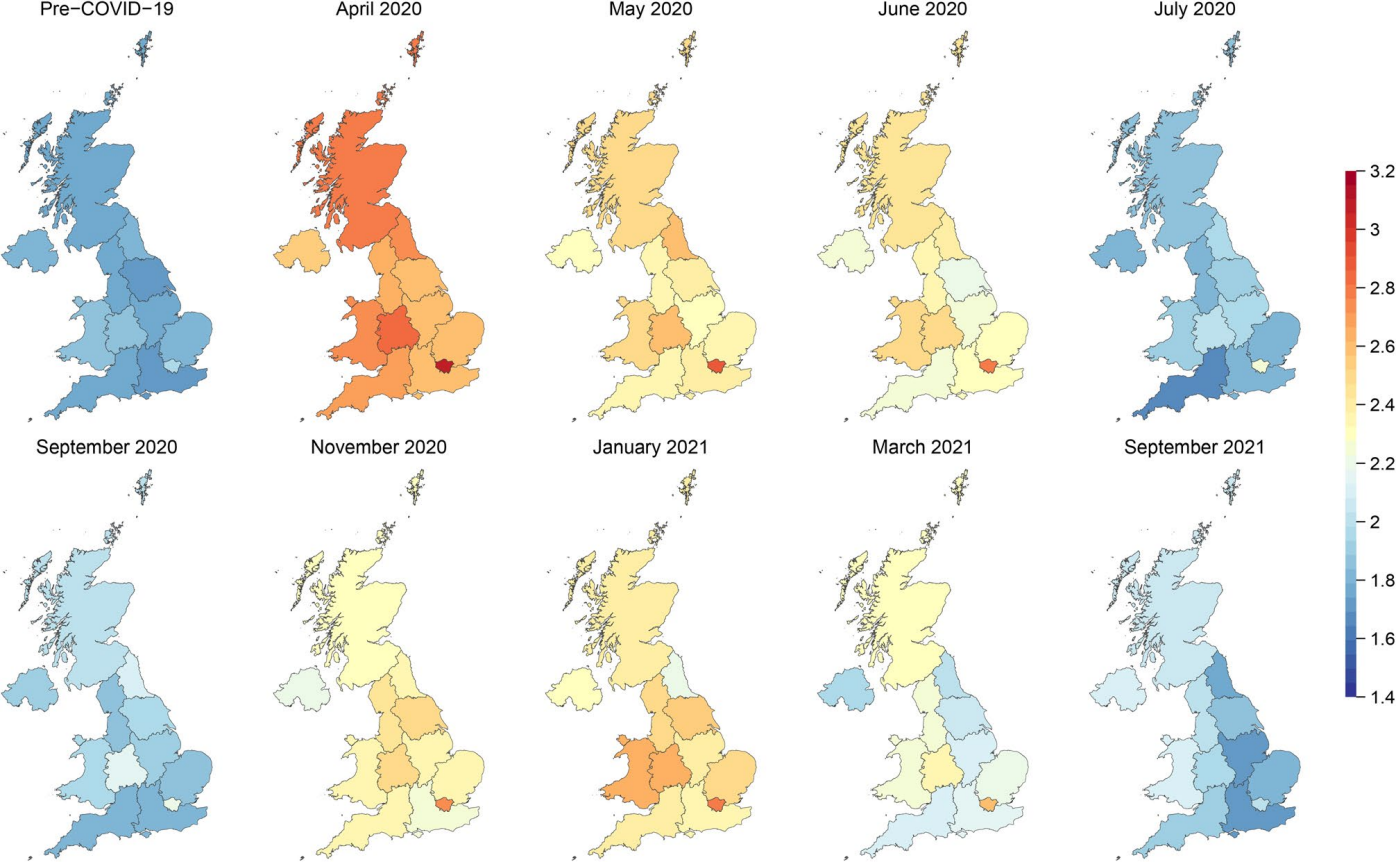
Mental health (GHQ Caseness score) across UK regions over time.

Figure 1 displays a choropleth map of regional average mental health (GHQ Caseness) in the UK. London’s residents experienced the worst subjective wellbeing for the entire duration of the dataset, including during the pre-pandemic period. Immediately after the declaration of the pandemic in March 2020, we observed a radical decline in people’s mental wellbeing. April 2020 is clearly the worst month. Generally, mental wellbeing showed an improvement as summer approached, which brought with it an easing of lockdown restrictions; this was followed by a deterioration again in winter with lockdowns reinstated. These panels in Fig. 1 portend our main results from the more sophisticated analysis—that increased mobility restrictions due to the lockdowns likely resulted in poorer mental health.

**Figure 2:**
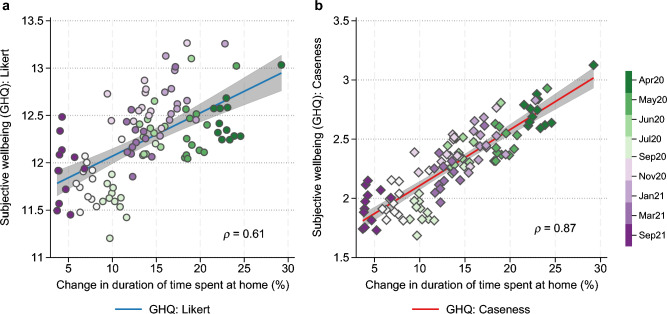
Mental health and mobility. Correlation between mobility and GHQ measures (GHQ Likert, GHQ Caseness) are monthly averages (9-wave) for each UK region. Marker colours represent the nine COVID-19 survey waves—earlier waves appear in green and later waves appear in purple. Shaded areas represent 95% confidence intervals of the linear fit.

We find a significant correlation between movement reduction and average mental health outcomes (prevalence of mental health issues). Figure 2 presents the linear fit between the mobility measure and mental health outcomes aggregated by regions and survey waves (averages). The correlation coefficients of regional-level mobility change and mental health are 0.61 and 0.87 (both *p* < 0.0001) for the two derivative measures of mental health (GHQ in a Likert scale, which ranges from 0 to 36 (Fig. 2a), and GHQ Caseness score, which ranges from 0 to 12 (Fig. 2b); in both cases, higher values correspond to worse mental health), respectively.

**Figure 3:**
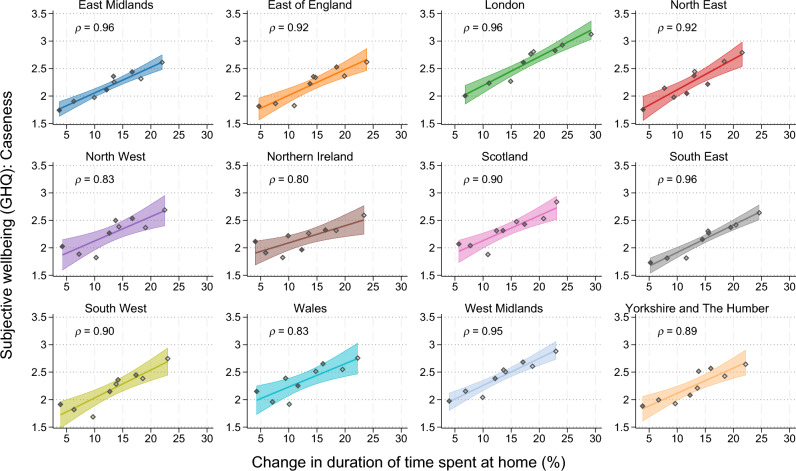
Relationship between GHQ Caseness score and mobility within region. Mental health (GHQ) is averaged over each survey wave for each UK region. Markers with darker colour represent later waves. Shaded areas represent 95% confidence intervals of the linear fit.

The largest change in the duration spent at home was experienced in the earlier parts of the pandemic (April and May 2020, for instance, have darker shades of green, which appear toward the right of each panel). This period is also associated with poorer mental health. A decrease in mobility at the societal level is also strongly associated with poorer mental health across all 12 regions. In Fig. 3, each dot represents the monthly average within each region, with the change in time spent at home (the horizontal axis) representing the deviation from the baseline in January 2020. Darker colours represent later waves.

Residents of London again display the strongest relationship (*ρ* = 0.97, *p* = 1.3 × 10^–5^) between the lockdown restrictions and mental health. For instance, people living in London experienced the most radical change in the duration spent at home—about 30% at its most extreme (in April 2020), which is associated with the worst average GHQ Caseness score in Fig. 3. Visually, the other regions are fairly similar to each other, with correlation ranging from 0.77 (Northern Ireland, *p* = 0.0154) to 0.97 (South East, *p* = 2.5 × 10^–5^). All 12 correlation coefficients are highly statistically significant at 5% (*p* < 0.05). At the LAD levels, positive correlations between residential mobility restrictions and mental health are evident (Fig. S3). On average, the correlation coefficients, when considering GHQ Caseness, reach 0.52 (with a median of ρ = 0.59). Among the 373 LADs, 346 (93%) of them exhibit positive correlations.

In Table 1, we show the main regression results using individual-level observations with the GHQ Caseness score as the outcome variable. As a benchmark, we begin with a bivariate model (Column 1) and subsequently include COVID test results and socio-demographic variables (Column 2), pre-existing mental health or long-term illness history (Column 3), COVID-19 prevalence and government stringency level in the last seven days (Column 4), region fixed effects (Column 5), wave fixed effects (Column 6), local authority district fixed effects (Column 7), and individual fixed effects (Column 8). The estimated relationship between movement reduction and mental health deterioration is statistically significant, at least at the 5% level. The main results are also robust to all specifications including the alternative derivative measure of mental health (i.e., Likert; Supplementary Table 2), using the past-14 days average for mobility, COVID-19 cases, and stringency measures (Supplementary Table 3), and clustering standard errors at the region/LAD or region/LAD-by-survey wave levels (Supplementary Table 4 and 5). We obtain robust and qualitatively similar results when we (1) cluster the standard errors at the household level, (2) use a subsample of individuals who completed all nine COVID-19 waves (40.5% of the full sample) or (3) exclude those subjects who did not participate in the latest pre-COVID wave (wave 10 in 2019; 5.72% of the full sample).

A 10-percentage-point increase in time spent at home (compared to pre-pandemic baseline January 2020) is associated with an average increase of 0.24–0.44 in GHQ Caseness score (which has a standard deviation of 3.35) [95% CI 0.08–0.47]. In standardised terms, a one standard deviation increase in residential mobility (about a 6.6 pp difference compared to pre-pandemic level) is associated with a 0.047–0.087 standard deviations increase in the GHQ Caseness score [95% CI 0.016–0.093]. The relationship holds true for GHQ on a Likert scale, albeit slightly weaker (Supplementary Table 2). In general, the relationship between the change in the time spent at home and mental distress is positive—that is, stay-at-home orders worsen mental wellbeing. The effect size is noticeable and comparable to or higher than the effect of other important observable characteristics, such as unemployment (+ 0.40 points in the GHQ Caseness score) or marriage (− 0.20 points). We also estimate the impact of lack of mobility on the single components of the GHQ Caseness score (Supplementary Fig. 4). Overall, decreasing mobility worsens all aspects of mental health, and the most noticeable effects are found on the inability to concentrate, to make decisions and to enjoy day-to-day activities. Other aspects, such as feeling worthless and under strain, were less affected.

The selection of adjustment variables is grounded on the health production function^56^, with current health—a capital good that depreciates over time—being a function of past investments in health. The set of independent variables included in the model is consistent with previous research^33,56,57^ and consists of a wide range of individual characteristics, such as indicators of socio-economic status and local area and family characteristics.

**Figure 4:**
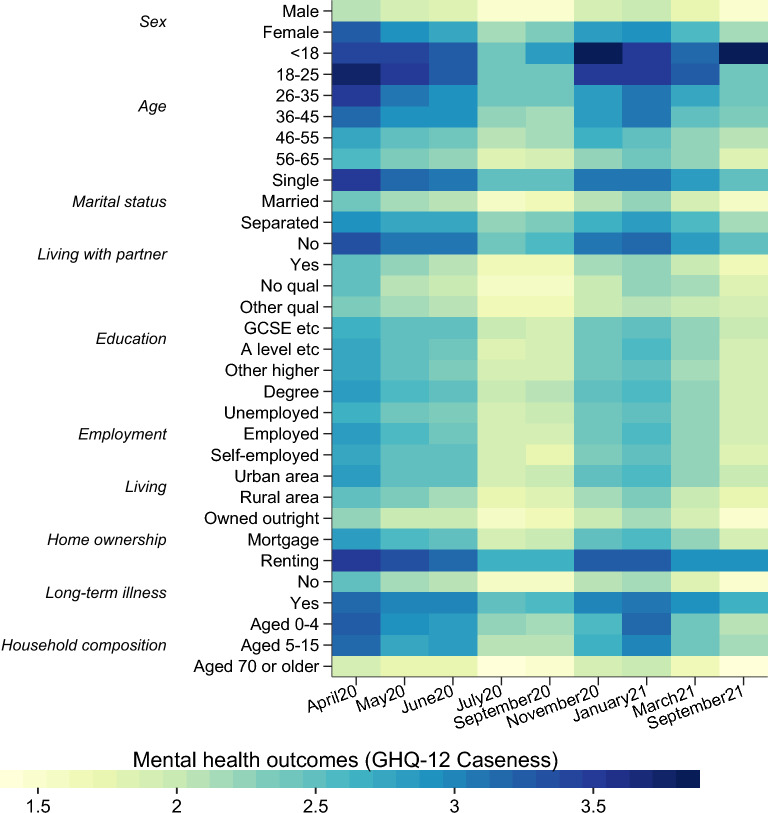
Mental health across different groups over time. Individual GHQ Caseness scores are averaged within groups across the nine waves of the COVID-19 Understanding Society.

In Fig. 4, we show how subjective wellbeing (measured as GHQ Caseness score) evolves over time for different socio-economic and demographic groups identified in the data. Notably, females, children, young adults, single households, and those with long-term illnesses have tracked much worse than other groups. Those renting their homes also experienced worse mental health. Nearly all these groups felt improvements in mental wellbeing between April 2020 and June 2020, which may reflect an adaptation to the “new normal” or public and private policies that improved individual and social wellbeing, or indeed the easing of lockdown measures. There was a notable deterioration of wellbeing around the time the second lockdown started in England (November 2020), but mental health also improved as the country transitions away from the day when the lockdown was introduced, like the evolution after the initial lockdown in March 2020.

In Tables 2 and 3, we interact the change in home duration with several different individual characteristics to examine their moderating roles. Recall from Fig. 4 that some groups experienced deeper declines in mental wellbeing—interacting the change in home duration with these variables allows us to demonstrate the kind of person that might be more adversely impacted by mobility restrictions, at least in terms of their effect on mental health. The set of control variables for these regressions with interaction terms are the same as those in Table 1.

In Table 2, the own-effect of the change in mobility is consistently positive, although lacking in statistical significance when we interact it with the gender of the respondent (Column 2). The interaction with the female variable, however, shows that women experienced a stronger decline in mental wellbeing than men over the period. Older respondents and those who were partnered were more resilient (Columns 1, 3, and 4). Finally, more educated individuals also experienced a worse decline in mental health (Column 5).

**Figure 5:**
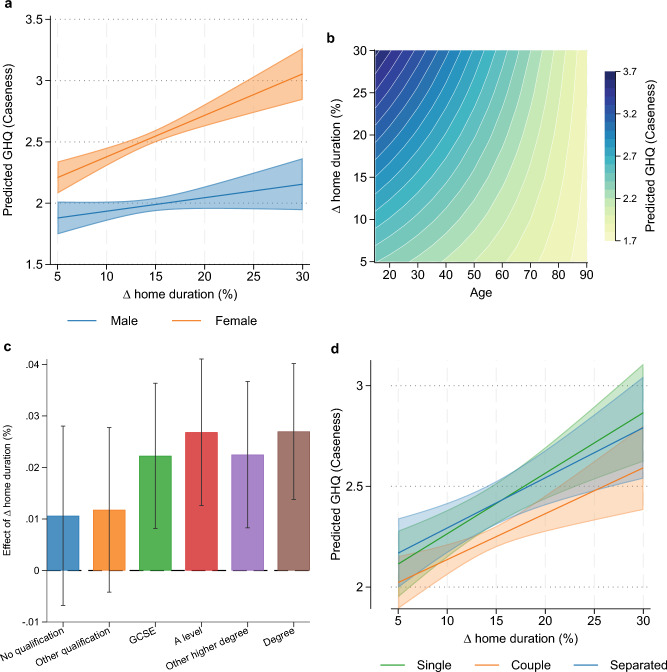
Heterogenous effect of lockdown on mental health across (**A**) gender, (**B**) age, (**C**) education, and (**D**) marital status groups. Estimated heterogenous effect of lockdown on mental health across groups were obtained from regression results in Table 2. Error bars (**C**) and shared area (**A** and **D**) represent 95% confidence intervals.

For ease of interpretation, we also graphically represent the estimation results of moderation in Fig. 5. For almost the entire range of the percentage change in time spent at home, women are worse off than men. The gradient is also consistent for the interaction with age: the larger the change in home duration, the worse off people are, but this relationship is much stronger for younger people than for older people.

In Table 3, we continue with the interactions of the change in time spent at home with the following moderators: living in an urban area, homeownership, employment status, a measure of pre-existing mental health (pre-COVID), and an indicator for having a long-term illness. Results reported in Table 3 show that those living in rural areas, the unemployed, and those with a long-term illness before COVID-19 started are less affected by the change in mobility. For example, the overall effect of mobility restriction on unemployed individuals is 0.0184 points on the GHQ score, and is calculated as the sum of 0.028 (effect of the increase in the proportion of time spent at home) and − 0.0096 (effect of the interaction between change in time spent at home and the binary unemployment variable). Similarly, individuals with better pre-pandemic mental health experienced a greater decline in wellbeing associated with residential stationarity.

**Figure 6:**
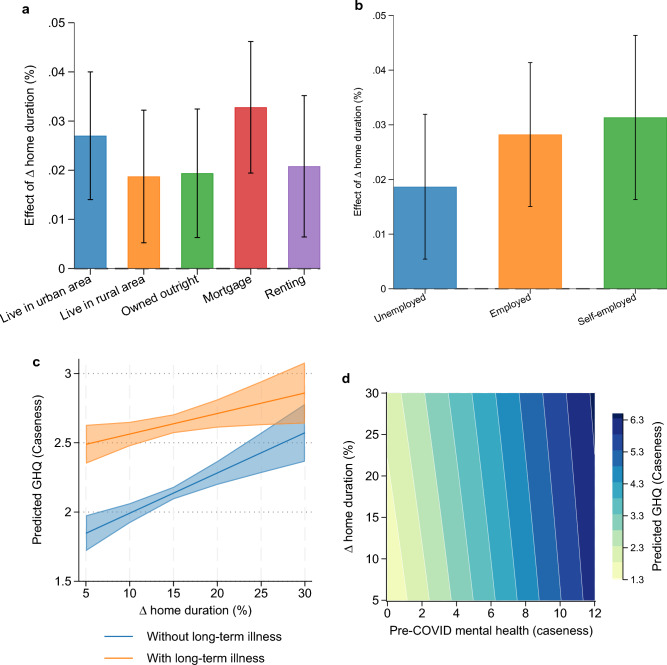
Heterogenous effect of lockdown on mental health across (**A**) homeownership and living area, (**B**) employment status during COVID-19, (**C**) long-standing illness or impairment, (**D**) pre-existing mental health issue groups. Estimated heterogenous effect of lockdown on mental health across groups were obtained from regression results in Table 3. Error bars (in panels a and b) and shared area (panel c) represent 95% confidence intervals.

Like Fig. 5, we also graphically represent these results in Fig. 6. Those who are paying off a mortgage and renting—perhaps because of increased financial pressure—show a larger deterioration in their mental health than those who own their domicile (Fig. 6a). The self-employed are also more adversely affected than those who are employed, and the unemployed are hardly affected at all (Fig. 6b). Across the range of the change in home duration, the effect of restrictions to mobility on mental health is less for those who have previous mental health issues or long-term illness (Fig. 6c and 6d).

**Figure 7:**
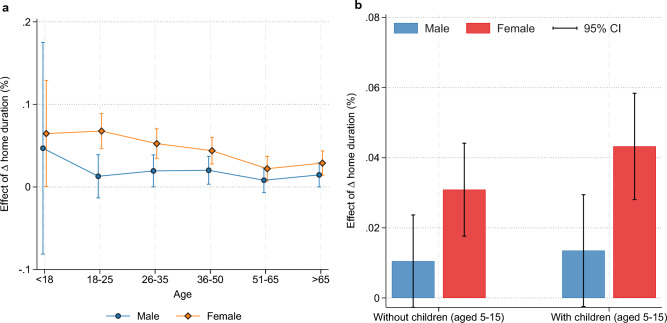
Three-way interactions of mobility, gender, and age group (**A**) and mobility, sex, and having a child aged 5–15 in the household (**B**). Error bars represent 95% confidence intervals.

In Fig. 7, we graphically represent the estimated coefficients on three-way interactions of mobility, gender, and age group (Fig. 7a) and mobility, gender, and an indicator for having a child aged 5–15 in the household (Fig. 7b). Younger women are more adversely affected than younger men, although the size of the differential declines as we move to older age groups. In addition, having a child in the household amplifies the negative relationship between mental health and being female during periods of increased mobility restrictions.

These estimates show how changes in average mobility impact the *average* mental health outcome. The main limitation of this approach is that, especially when we interpret interaction effects, the underlying assumption is that the movement change is of the same magnitude for the whole population. This is unlikely to be the case, as older people or people with long-term illnesses were less mobile even before the pandemic started. Therefore, caution should be used when interpreting these estimates, as this type of measurement error could create attenuation bias in the results, which could overestimate the effects for groups of individuals whose movement change is less than the population average, and underestimate the effects for those whose mobility was higher.

However, the negative impact of lack of mobility may arise from several sources apart from restrictions to individuals’ movement. For example, it is possible that the lack of mobility in society, which is in turn reflected in a decrease in available services and overall social interaction, may have a negative impact on individual wellbeing, in addition to the effects due to the restrictions on the individual mobility.

## Discussion

We show a significant association between restrictions to mobility and the worsening of individuals’ mental wellbeing. These results have considerable implications for the management of future pandemics. Since the start of the pandemic, evidence regarding the adverse impact of lockdowns on subjective wellbeing has accumulated in the scientific literature, including the current study. Although the evidence that lockdowns suppress the transmission of the disease is clear, it must be balanced against the real costs associated with mental health deterioration when physical human contact is limited via mobility restrictions.

We combined a robust measure of mental health—the GHQ Caseness score—with data from Google on mobility restrictions to track how mental wellbeing evolved during periods of lockdown and easing of restrictions in the UK. We demonstrated that the decline in mental health is significant, and that certain groups experienced significantly sharper deteriorations in mental health than others. Particularly noteworthy are the gender-based differences: women experienced a greater decline in mental wellbeing associated with residential stationarity than men, and this is especially pronounced if there are small children in the household, perhaps reflecting the increased burden of domestic or at-home childcare faced by women when schools pivot to remote learning. Households that are relatively more financially insecure are also at risk for further mental health distress, as well as those who are not partnered and may not have someone with whom to share the burdens of lockdown.

Combining survey and digital data enables us to investigate a common problem in evaluation research: namely, that relevant outcome data (in this case, information on mental health) is collected retrospectively to a shock through ad hoc surveys. As a result, one learns about the effect of current restrictions with a lag, which can vary from weeks to months or years (health-related questions often appear in annual surveys) and with limited or ex-post information on pre-shock conditions. This delay, in turn, informs on the problem only when it has already grown, possibly with tragic consequences such as self-harm, suicides, and domestic violence, to name a few. In contrast, by combining daily search data with the concurrent outcome of interest, we shed light on whether digital data offer insightful societal and policy feedback to evaluate or tune policies in almost real-time. For example, smartphones generate instant tracking via a digital human footprint of society^12–14^ and hence present almost real-time data on the variables, like mobility, that policymakers intend to affect (e.g., through lockdowns) without the organisational and operational challenges to collect such information via surveys.

Highlighting the potential of enhancing survey data with high-frequency data fills a gap in the existing literature. This issue has received only limited attention within social sciences, as these have tended to focus on either macro-based and cultural indicators rather than on mobility broadly defined—that is, including the analysis of labour, employment, and mobility across differing occupations^58^, or behavioural aspects around mobility such as risk^59^, trust^60^ or personality characteristics^61^. This status quo encases social science research within a narrower focus than what is required to understand the complex relationship between mobility and mental health^34,62,63^—notwithstanding health sciences research pointing to the relationship between mobility behaviours and mental health outcomes^64–66^.

From a policy perspective—when lockdowns are unavoidable—it makes sense to attempt to limit the decline in mental health for the population under lockdown. Governments can invest in the capacity to manage deteriorating mental health via increased funding for psychological support and counselling through telemedicine or online consultations, as well as the increased guarantee of job security and the provision of financial aid when people are unable to work. Schools can remain open for as long as possible to allow parents to work at home without having to attend to childcare and to reduce the disruption in student learning, which may have longer-term impacts over the child’s life course and the mental wellbeing of the parents.

Moreover, as Snowden^67^ points out in his historical overview of pandemics, “major epidemics caught authorities unprepared, leading to confusion, chaos, and improvision” (p. 77). Under such circumstances, policy indicators via survey data are often not fast enough to guide effective responses. By the time such data are generated, decisions are already made that can have long-lasting effects on society. The push towards Big Data allows the use of high-frequency data in the policy realm^68^. In addition to helpline call volume data^43^, real-time mobility data as an information source also provides a good general indicator for the state of public mental health during a pandemic, which can be added to the toolset of ambient and passive sensing wellbeing monitoring^69–74^. Although such high-frequency data are not fine-grained enough for some purposes, the relationship observed here encourages further consideration of tapping into various sources of high-frequency data to make informed policy decisions. In times of crisis where lagged availability can produce societal costs, it is particularly important for decision makers to remain open and consider information that can be gathered from alternative measures to improve the agility and adaptiveness of policy responses.

### Supplementary Information

Supplementary Information.

## Data Availability

Data from the Understanding Society are available through the UK Data Service, providing granted research access (Institute for Social and Economic Research, 2022). Mobility data were downloaded from Google’s COVID-19 Community Mobility Report. UK COVID-19 epidemiological data were collect-ed from the UK Health Security Agency. Subnational UK COVID-19 Stringency Index were obtained from the Oxford Coronavirus Government Response Tracker (OxCGRT). Code used to analyze data and generate results are available from OSF https://osf.io/hg6e4/.
