# Supplementary material for: Residential mobility restrictions and adverse mental health outcomes during the COVID-19 pandemic in the UK
Source: Sci Rep. 2024 Jan 20;14:1790. doi: 10.1038/s41598-024-51854-6 (PMC10799952; doi:10.1038/s41598-024-51854-6)
Supplement: Supplementary file 1 — Supplementary Information. [file 41598_2024_51854_MOESM1_ESM.docx]

**Supplementary Information for**

Residential mobility restrictions and adverse mental health outcomes during the COVID-19 pandemic in the UK

Ho Fai Chan, Zhiming Cheng, Silvia Mendolia, Alfredo R. Paloyo, Massimiliano Tani, Damon Proulx, David A. Savage, Benno Torgler

Correspondence author: Ho Fai Chan

Email: hofai.chan@qut.edu.au

**This PDF file includes:**

Supplementary Notes

Figures S1 to S3

Tables S1 to S5

**Supplementary Notes.**

**List of questions in the General Health Questionnaire**

The next questions are about how you have been feeling over the last few weeks.

1. Have you recently been able to concentrate on whatever you're doing?
2. Have you recently lost much sleep over worry?
3. Have you recently felt that you were playing a useful part in things?
4. Have you recently felt capable of making decisions about things?
5. Have you recently felt constantly under strain?
6. Have you recently felt you couldn't overcome your difficulties?
7. Have you recently been able to enjoy your normal day-to-day activities?
8. Have you recently been able to face up to problems?
9. Have you recently been feeling unhappy or depressed?
10. Have you recently been losing confidence in yourself?
11. Have you recently been thinking of yourself as a worthless person?
12. Have you recently been feeling reasonably happy, all things considered?

Possible answers are: Not at all; No more than usual; Rather more than usual; Much more than usual.

The GHQ Caseness score ranges from 0 to 12 and is constructed summing the number of times the individual places herself/himself in the most distressed category.

**
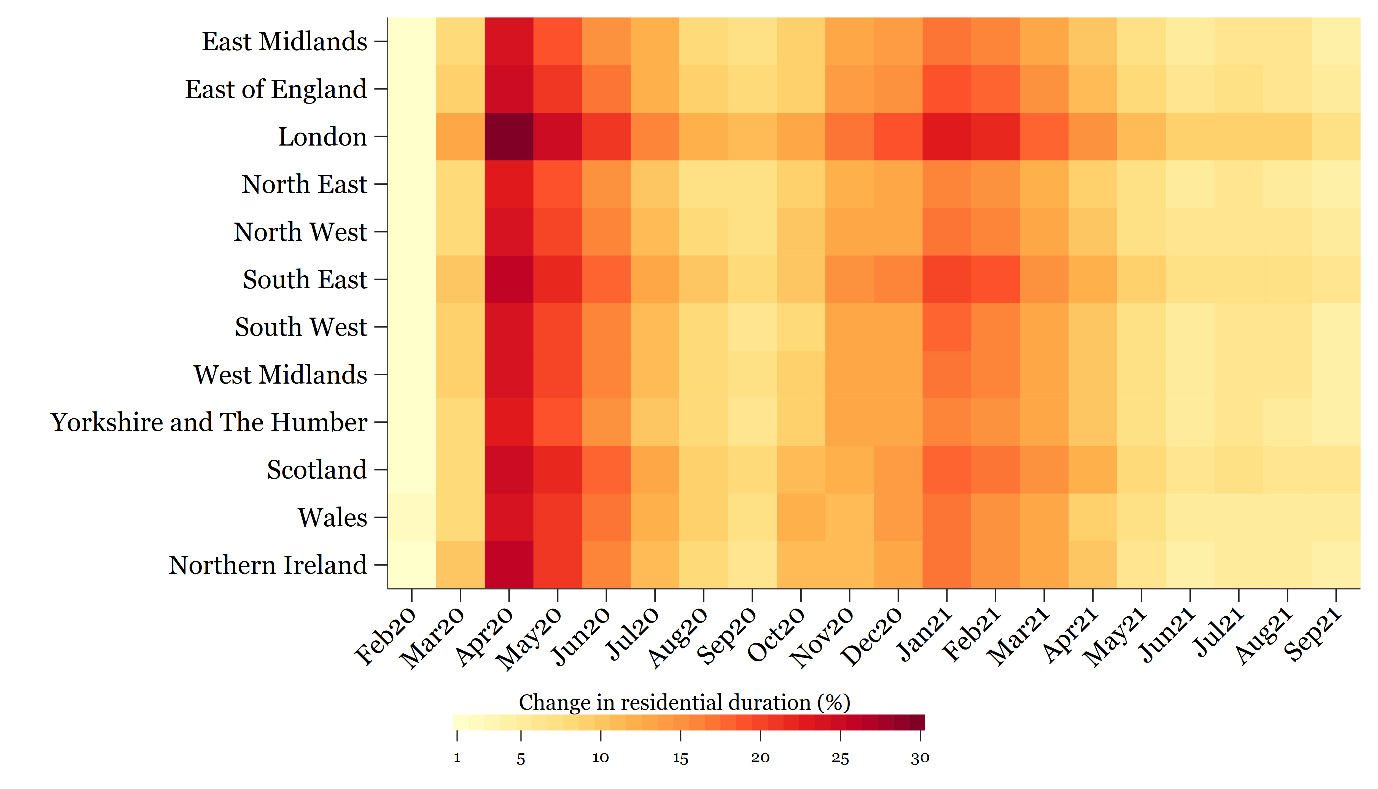
**

**Figure S1. Mobility changes of the 12 UK regions from February 2020 to September 2021.**

**
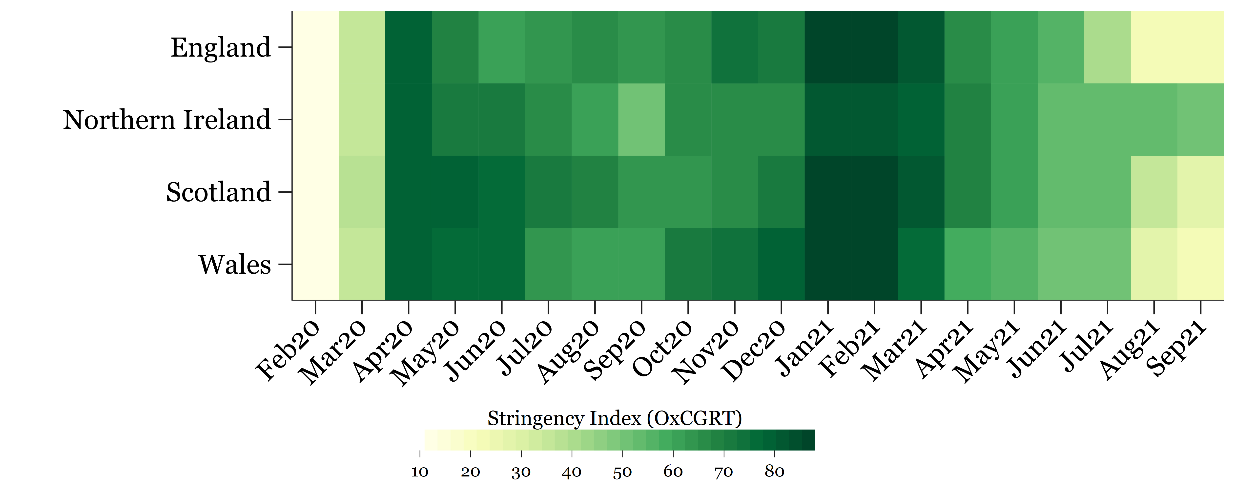
**

**Figure S2. Government stringency level (OxCGRT) of UK countries from February 2020 to September 2021.**


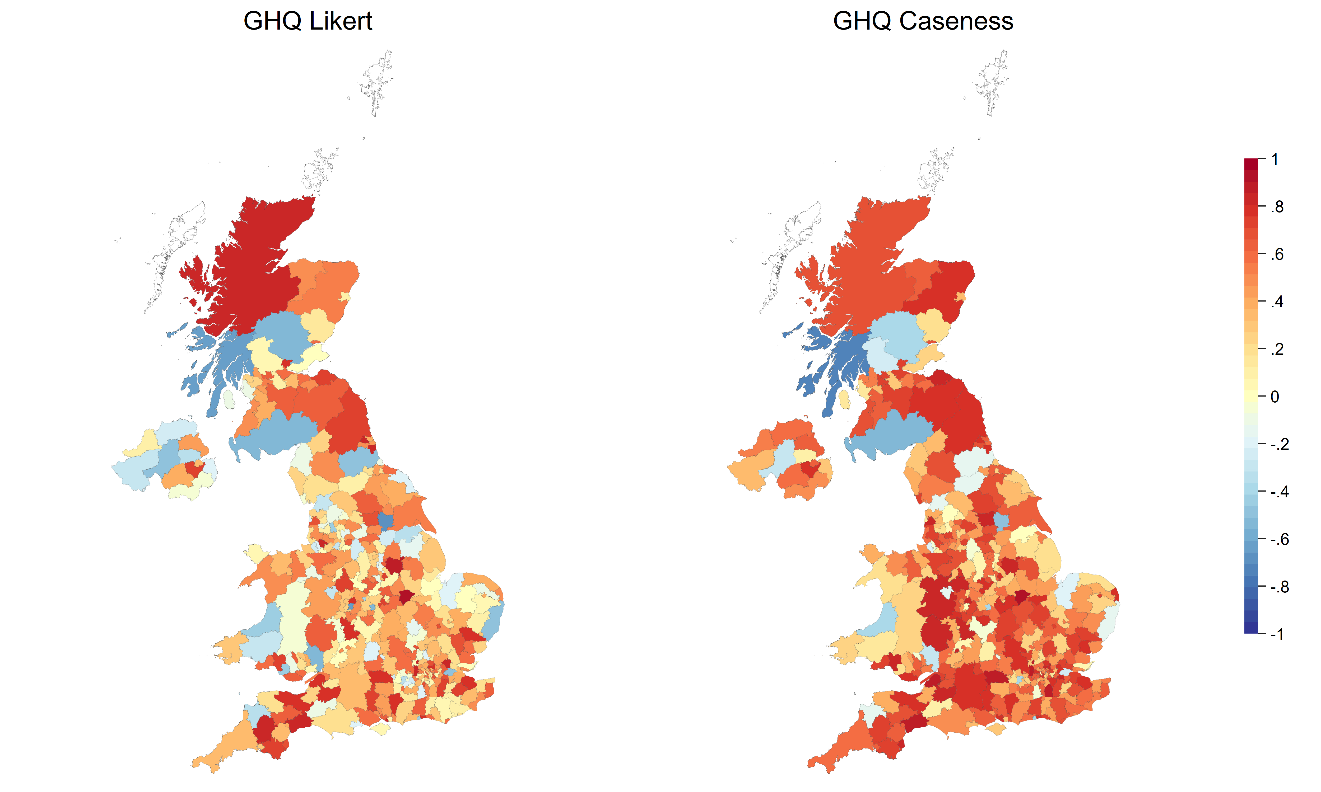
 **Figure S3. Correlations between** **residential mobility restrictions and mental health at the Local Authority Districts level.**


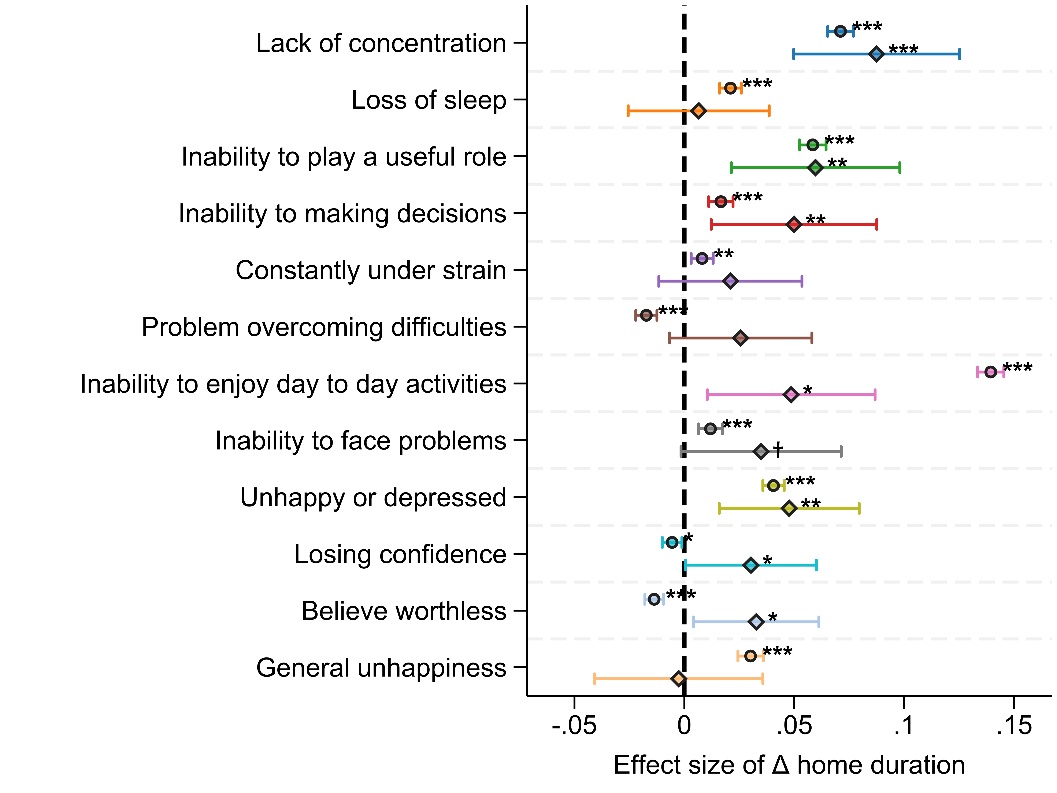


**Figure S4. Effect of movement restrictions on the 12 GHQ items.** Each marker represents the (standardized) effect size of change in the duration of time spent at home on the GHQ item obtained from simple regressions (circle, analogous to Model 1 in Table 1) and regressions with time-varying variables and region, wave, and individual fixed effects (diamond, analogous to Model 7 in Table 1). † *p* < .10; * *p* < .05; ** *p* < .01; *** *p* < .001.

**Table S1. Sample descriptive statistics**

|  | *Observation* | | | | | *Individual* | | | | |
| --- | --- | --- | --- | --- | --- | --- | --- | --- | --- | --- |
|  | N_obs_ | Mean/% | SD | Min | Max | N_ind_ | Mean/% | SD | Min | Max |
| Mental wellbeing (GHQ): Likert | 117950 | 12.23 | 5.93 | 0 | 36 | 18625 | 12.46 | 5.24 | 0 | 36 |
| Mental wellbeing (GHQ): Caseness | 117950 | 2.30 | 3.35 | 0 | 12 | 18625 | 2.44 | 2.84 | 0 | 12 |
| COVID-19 positive | 122826 | 14.93% |  |  |  | 19763 | 12.67% |  |  |  |
| Age | 122826 | 53.27 | 16.50 | 15 | 114 | 19763 | 50.27 | 17.15 | 15 | 114 |
| *Gender* | 122812 |  |  |  |  | 19761 |  |  |  |  |
| Male |  | 41.40% |  |  |  |  | 41.92% |  |  |  |
| Female |  | 58.60% |  |  |  |  | 58.08% |  |  |  |
| *Marital status* | 120489 |  |  |  |  | 19208 |  |  |  |  |
| Single |  | 23.38% |  |  |  |  | 26.87% |  |  |  |
| Married/civil partnership |  | 61.22% |  |  |  |  | 58.73% |  |  |  |
| Separated/divorced/widowed |  | 15.40% |  |  |  |  | 14.40% |  |  |  |
| Living with partner | 122825 | 69.96% |  |  |  | 19763 | 69.47% |  |  |  |
| *Education* | 120512 |  |  |  |  | 19247 |  |  |  |  |
| No qualification |  | 4.20% |  |  |  |  | 4.31% |  |  |  |
| Other qualification |  | 6.76% |  |  |  |  | 6.70% |  |  |  |
| GCSE |  | 17.84% |  |  |  |  | 18.34% |  |  |  |
| A level |  | 20.58% |  |  |  |  | 21.27% |  |  |  |
| Other higher degree |  | 13.97% |  |  |  |  | 13.57% |  |  |  |
| Degree |  | 36.66% |  |  |  |  | 35.82% |  |  |  |
| *Living area* | 121347 |  |  |  |  | 19416 |  |  |  |  |
| Urban |  | 73.92% |  |  |  |  | 74.91% |  |  |  |
| Rural |  | 26.08% |  |  |  |  | 25.09% |  |  |  |
| *Housing status* | 118468 |  |  |  |  | 18819 |  |  |  |  |
| Owned outright |  | 41.86% |  |  |  |  | 37.36% |  |  |  |
| Mortgage |  | 40.41% |  |  |  |  | 42.31% |  |  |  |
| Renting |  | 17.73% |  |  |  |  | 20.34% |  |  |  |
| *Employment* | 121128 |  |  |  |  | 18713 |  |  |  |  |
| Unemployed |  | 41.49% |  |  |  |  | 38.67% |  |  |  |
| Employed |  | 48.96% |  |  |  |  | 51.42% |  |  |  |
| Self-employed |  | 9.55% |  |  |  |  | 9.91% |  |  |  |
| *Household composition* |  |  |  |  |  |  |  |  |  |  |
| Aged 0-4 | 122825 | 0.10 | 0.35 | 0 | 2 | 19763 | 0.12 | 0.39 | 0 | 2 |
| Aged 5-15 | 122825 | 0.32 | 0.69 | 0 | 3 | 19763 | 0.39 | 0.75 | 0 | 3 |
| Aged 70 or older | 122825 | 0.15 | 0.37 | 0 | 2 | 19763 | 0.15 | 0.39 | 0 | 2 |
| Pre-COVID GHQ (Likert) | 119195 | 11.22 | 5.44 | 0 | 36 | 18928 | 11.37 | 5.56 | 0 | 36 |
| Long-standing illness or impairment | 121208 | 0.35 | 0.48 | 0 | 1 | 19391 | 0.33 | 0.47 | 0 | 1 |

*Notes*: Mental wellbeing and COVID-19 positive indicator were averaged across all waves for summary statistics on the individual level.

**Table S2. Mental wellbeing (GHQ): Likert**

|  | (1) | (2) | (3) | (4) | (5) | (6) | (7) | (8) |
| --- | --- | --- | --- | --- | --- | --- | --- | --- |
| Change in duration of time spent at home (%) | 0.036^***^ | 0.040^***^ | 0.039^***^ | 0.028^***^ | 0.028^***^ | 0.035^**^ | 0.040^**^ | 0.042^**^ |
|  | (0.00211) | (0.00217) | (0.00217) | (0.00309) | (0.00314) | (0.0111) | (0.0137) | (0.0136) |
| COVID-19 positive |  | 0.51^***^ | 0.48^***^ | 0.34^***^ | 0.35^***^ | 0.19^**^ | 0.19^**^ | 0.18^**^ |
|  |  | (0.0578) | (0.0566) | (0.0589) | (0.0590) | (0.0608) | (0.0611) | (0.0674) |
| Age |  | -0.039^***^ | -0.030^***^ | -0.031^***^ | -0.031^***^ | -0.033^***^ | -0.033^***^ |  |
|  |  | (0.00373) | (0.00330) | (0.00336) | (0.00337) | (0.00338) | (0.00340) |  |
| Female |  | 1.33^***^ | 0.84^***^ | 0.83^***^ | 0.83^***^ | 0.83^***^ | 0.83^***^ |  |
|  |  | (0.0752) | (0.0643) | (0.0653) | (0.0653) | (0.0653) | (0.0649) |  |
| *Marital status* |  |  |  |  |  |  |  |  |
| Married/civil partnership |  | -0.52^***^ | -0.27^*^ | -0.22^*^ | -0.21^†^ | -0.20^†^ | -0.18^†^ |  |
|  |  | (0.124) | (0.108) | (0.110) | (0.110) | (0.110) | (0.111) |  |
| Separated/divorced/widowed |  | 0.039 | -0.0095 | 0.030 | 0.034 | 0.066 | 0.062 |  |
|  |  | (0.156) | (0.131) | (0.133) | (0.133) | (0.133) | (0.133) |  |
| Living with partner |  | -0.47^***^ | -0.35^***^ | -0.40^***^ | -0.40^***^ | -0.38^***^ | -0.38^***^ | -0.27^**^ |
|  |  | (0.0807) | (0.0765) | (0.0786) | (0.0787) | (0.0787) | (0.0792) | (0.0974) |
| *Education* |  | -0.074 | -0.29 | -0.29 | -0.29 | -0.26 | -0.25 |  |
| No qualification |  | (0.215) | (0.174) | (0.178) | (0.178) | (0.178) | (0.181) |  |
|  |  | -0.13 | -0.15 | -0.19 | -0.19 | -0.18 | -0.19 |  |
| Other qualification |  | (0.173) | (0.143) | (0.144) | (0.144) | (0.144) | (0.144) |  |
|  |  | -0.033 | 0.055 | 0.061 | 0.055 | 0.051 | 0.063 |  |
| A level |  | (0.124) | (0.105) | (0.107) | (0.107) | (0.107) | (0.107) |  |
|  |  | 0.056 | 0.17 | 0.20^†^ | 0.19^†^ | 0.19^†^ | 0.20^†^ |  |
| Other higher degree |  | (0.135) | (0.113) | (0.115) | (0.115) | (0.115) | (0.115) |  |
|  |  | 0.011 | 0.16^†^ | 0.17^†^ | 0.17^†^ | 0.17^†^ | 0.18^†^ |  |
| Degree |  | (0.110) | (0.0935) | (0.0952) | (0.0958) | (0.0961) | (0.0973) |  |
|  |  | -0.20^*^ | -0.11 | -0.078 | -0.056 | -0.079 | -0.059 |  |
| Live in rural area |  | (0.0835) | (0.0711) | (0.0727) | (0.0750) | (0.0752) | (0.0909) |  |
| *Housing status* |  |  |  |  |  |  |  |  |
| Mortgage |  | 0.62^***^ | 0.23^**^ | 0.22^*^ | 0.22^*^ | 0.20^*^ | 0.20^*^ |  |
|  |  | (0.0972) | (0.0836) | (0.0853) | (0.0854) | (0.0855) | (0.0869) |  |
| Renting |  | 1.64^***^ | 0.66^***^ | 0.65^***^ | 0.65^***^ | 0.63^***^ | 0.62^***^ |  |
|  |  | (0.129) | (0.107) | (0.109) | (0.110) | (0.110) | (0.110) |  |
| *Employment* |  |  |  |  |  |  |  |  |
| Unemployed |  | 0.93^***^ | 0.65^***^ | 0.63^***^ | 0.63^***^ | 0.63^***^ | 0.64^***^ | 0.80^***^ |
|  |  | (0.0868) | (0.0804) | (0.0840) | (0.0840) | (0.0843) | (0.0847) | (0.125) |
| Self-employed |  | 0.27^*^ | 0.32^**^ | 0.33^**^ | 0.32^**^ | 0.32^**^ | 0.30^**^ | 0.13 |
|  |  | (0.113) | (0.104) | (0.104) | (0.104) | (0.105) | (0.105) | (0.170) |
| *Household composition* |  |  |  |  |  |  |  |  |
| Aged 0-4 |  | -0.035 | 0.061 | 0.064 | 0.064 | 0.070 | 0.065 |  |
|  |  | (0.0901) | (0.0840) | (0.0843) | (0.0843) | (0.0843) | (0.0846) |  |
| Aged 5-15 |  | 0.035 | 0.013 | 0.036 | 0.035 | 0.040 | 0.040 |  |
|  |  | (0.0524) | (0.0476) | (0.0486) | (0.0486) | (0.0485) | (0.0489) |  |
| Aged 70 or older |  | -0.18^*^ | -0.13^†^ | -0.079 | -0.078 | -0.063 | -0.061 |  |
|  |  | (0.0754) | (0.0703) | (0.0718) | (0.0718) | (0.0719) | (0.0722) |  |
| Pre-COVID GHQ (Likert) |  |  | 0.47^***^ | 0.47^***^ | 0.47^***^ | 0.47^***^ | 0.47^***^ |  |
|  |  |  | (0.00823) | (0.00839) | (0.00840) | (0.00840) | (0.00834) |  |
| Long-standing illness or impairment |  |  | 0.70^***^ | 0.74^***^ | 0.74^***^ | 0.75^***^ | 0.73^***^ |  |
|  |  |  | (0.0719) | (0.0729) | (0.0730) | (0.0731) | (0.0731) |  |
| Case per 1,000 people |  |  |  | 11.3^***^ | 11.3^***^ | 2.06 | 1.46 | 1.97 |
|  |  |  |  | (1.205) | (1.230) | (1.622) | (1.685) | (1.673) |
| Stringency index |  |  |  | 0.0089^***^ | 0.0089^***^ | 0.0076^†^ | 0.0077^†^ | 0.0087^†^ |
|  |  |  |  | (0.000947) | (0.000956) | (0.00452) | (0.00458) | (0.00454) |
| Constant | 11.9^***^ | 12.7^***^ | 7.09^***^ | 6.64^***^ | 6.57^***^ | 6.45^***^ | 5.55^***^ | 10.4^***^ |
|  | (0.0481) | (0.231) | (0.219) | (0.226) | (0.248) | (0.470) | (0.964) | (0.446) |
| Region FE | No | No | No | No | Yes | Yes | No | Yes |
| Wave FE | No | No | No | No | No | Yes | Yes | Yes |
| Individual FE | No | No | No | No | No | No | No | Yes |
| LAD FE | No | No | No | No | No | No | Yes | No |
| Observations | 116513 | 110840 | 109268 | 101236 | 101236 | 101236 | 101236 | 108001 |
| Individuals (cluster) | 18517 | 17361 | 17049 | 16662 | 16662 | 16662 | 16662 | 18073 |
| *R^2^-within* | 0.004 | 0.006 | 0.006 | 0.009 | 0.009 | 0.013 | 0.013 | 0.013 |
| *R^2^-between* | 0.001 | 0.082 | 0.332 | 0.334 | 0.335 | 0.335 | 0.350 | 0.003 |
| *R^2^-overall* | 0.002 | 0.063 | 0.254 | 0.256 | 0.256 | 0.257 | 0.267 | 0.004 |
| Prob. > *F.* | 0.000 | 0.000 | 0.000 | 0.000 | 0.000 | 0.000 | 0.000 | 0.000 |

*Notes*: GLS regressions. Reference group: *Male, Single, Not living with a partner, Live in Urban area, Owned outright, Employed.* Standard errors (clustered at individual level) in parentheses. † *p* < .10; * *p* < .05; ** *p* < .01; *** *p* < .001.

**Table S3. Using past 14 days average instead of 7 days, for GHQ Caseness score**

|  | (1) | (2) | (3) | (4) | (5) | (6) | (7) | (8) |
| --- | --- | --- | --- | --- | --- | --- | --- | --- |
| Change in duration of time spent at home (%) | 0.041*** | 0.042*** | 0.042*** | 0.040*** | 0.040*** | 0.024*** | 0.024** | 0.025** |
|  | (0.00121) | (0.00126) | (0.00126) | (0.00175) | (0.00178) | (0.00673) | (0.00874) | (0.00862) |
| COVID-19 positive |  | 0.22*** | 0.21*** | 0.17*** | 0.17*** | 0.14*** | 0.14*** |  |
|  |  | (0.0345) | (0.0340) | (0.0351) | (0.0352) | (0.0363) | (0.0365) |  |
| Age |  | -0.018*** | -0.014*** | -0.015*** | -0.015*** | -0.015*** | -0.015*** |  |
|  |  | (0.00203) | (0.00187) | (0.00192) | (0.00192) | (0.00192) | (0.00194) |  |
| Female |  | 0.75*** | 0.56*** | 0.55*** | 0.55*** | 0.55*** | 0.55*** |  |
|  |  | (0.0407) | (0.0368) | (0.0375) | (0.0375) | (0.0375) | (0.0372) |  |
| *Marital status* |  |  |  |  |  |  |  |  |
| Married/civil partnership |  | -0.31*** | -0.19** | -0.17** | -0.16* | -0.16* | -0.15* |  |
|  |  | (0.0681) | (0.0623) | (0.0635) | (0.0635) | (0.0635) | (0.0640) |  |
| Separated/divorced/widowed |  | 0.014 | -0.0091 | 0.0060 | 0.0031 | 0.0046 | 0.0087 |  |
|  |  | (0.0849) | (0.0752) | (0.0765) | (0.0766) | (0.0766) | (0.0771) |  |
| Living with partner |  | -0.29*** | -0.21*** | -0.23*** | -0.23*** | -0.23*** | -0.23*** | -0.17** |
|  |  | (0.0459) | (0.0442) | (0.0455) | (0.0455) | (0.0455) | (0.0458) | (0.0581) |
| *Education* |  | -0.24* | -0.31** | -0.29** | -0.29** | -0.29** | -0.28** |  |
| No qualification |  | (0.115) | (0.0986) | (0.101) | (0.101) | (0.101) | (0.103) |  |
|  |  | -0.12 | -0.14† | -0.16* | -0.16† | -0.16† | -0.17* |  |
| Other qualification |  | (0.0927) | (0.0803) | (0.0810) | (0.0811) | (0.0811) | (0.0812) |  |
|  |  | -0.014 | 0.0052 | 0.014 | 0.011 | 0.011 | 0.012 |  |
| A level |  | (0.0670) | (0.0598) | (0.0612) | (0.0612) | (0.0612) | (0.0612) |  |
|  |  | 0.063 | 0.083 | 0.089 | 0.085 | 0.089 | 0.086 |  |
| Other higher degree |  | (0.0736) | (0.0654) | (0.0667) | (0.0667) | (0.0668) | (0.0666) |  |
|  |  | 0.16** | 0.16** | 0.17** | 0.17** | 0.18** | 0.17** |  |
| Degree |  | (0.0598) | (0.0537) | (0.0548) | (0.0552) | (0.0553) | (0.0560) |  |
|  |  | -0.057 | -0.030 | -0.0097 | -0.0055 | -0.024 | 0.000088 |  |
| Live in rural area |  | (0.0454) | (0.0408) | (0.0419) | (0.0433) | (0.0435) | (0.0523) |  |
| *Housing status* |  |  |  |  |  |  |  |  |
| Mortgage |  | 0.28*** | 0.13** | 0.12* | 0.12* | 0.12* | 0.12* |  |
|  |  | (0.0532) | (0.0482) | (0.0493) | (0.0493) | (0.0493) | (0.0501) |  |
| Renting |  | 0.77*** | 0.34*** | 0.33*** | 0.34*** | 0.34*** | 0.32*** |  |
|  |  | (0.0701) | (0.0620) | (0.0632) | (0.0635) | (0.0635) | (0.0636) |  |
| *Employment* |  |  |  |  |  |  |  |  |
| Unemployed |  | 0.54*** | 0.38*** | 0.38*** | 0.38*** | 0.38*** | 0.39*** | 0.46*** |
|  |  | (0.0475) | (0.0451) | (0.0470) | (0.0470) | (0.0471) | (0.0473) | (0.0710) |
| Self-employed |  | 0.22*** | 0.22*** | 0.23*** | 0.23*** | 0.23*** | 0.22*** | 0.094 |
|  |  | (0.0611) | (0.0576) | (0.0581) | (0.0581) | (0.0581) | (0.0586) | (0.0975) |
| *Household composition* |  |  |  |  |  |  |  |  |
| Aged 0-4 |  | 0.024 | 0.075 | 0.063 | 0.062 | 0.062 | 0.059 |  |
|  |  | (0.0514) | (0.0495) | (0.0505) | (0.0505) | (0.0505) | (0.0507) |  |
| Aged 5-15 |  | 0.059* | 0.058* | 0.067* | 0.066* | 0.067* | 0.066* |  |
|  |  | (0.0291) | (0.0273) | (0.0281) | (0.0281) | (0.0281) | (0.0283) |  |
| Aged 70 or older |  | -0.100* | -0.069† | -0.052 | -0.050 | -0.052 | -0.050 |  |
|  |  | (0.0436) | (0.0415) | (0.0427) | (0.0427) | (0.0427) | (0.0428) |  |
| Pre-COVID GHQ (caseness) |  |  | 0.38*** | 0.38*** | 0.38*** | 0.38*** | 0.38*** |  |
|  |  |  | (0.00829) | (0.00845) | (0.00845) | (0.00845) | (0.00837) |  |
| Long-standing illness or impairment |  |  | 0.49*** | 0.51*** | 0.51*** | 0.51*** | 0.50*** |  |
|  |  |  | (0.0415) | (0.0423) | (0.0423) | (0.0423) | (0.0422) |  |
| Case per 1,000 people |  |  |  | 3.64*** | 3.55*** | 0.98 | 0.84 | 1.09 |
|  |  |  |  | (0.619) | (0.630) | (0.817) | (0.837) | (0.831) |
| Stringency index |  |  |  | 0.0020*** | 0.0019** | 0.0044 | 0.0046 | 0.0051† |
|  |  |  |  | (0.000567) | (0.000572) | (0.00279) | (0.00284) | (0.00281) |
| Constant | 1.77*** | 2.05*** | 1.20*** | 1.09*** | 1.05*** | 1.26*** | 1.03* | 1.57*** |
|  | (0.0274) | (0.126) | (0.115) | (0.120) | (0.134) | (0.278) | (0.483) | (0.273) |
| Region FE | No | No | No | No | Yes | Yes | No | No |
| LAD FE | No | No | No | No | No | No | Yes | No |
| Wave FE | No | No | No | No | No | Yes | Yes | Yes |
| Individual FE | No | No | No | No | No | No | No | Yes |
| Observations | 116513 | 110840 | 109268 | 101236 | 101236 | 101236 | 101236 | 108001 |
| Individuals (cluster) | 18517 | 17361 | 17049 | 16662 | 16662 | 16662 | 16662 | 18073 |
| *R^2^-within* | 0.015 | 0.016 | 0.016 | 0.017 | 0.017 | 0.018 | 0.019 | 0.018 |
| *R^2^-between* | 0.004 | 0.078 | 0.259 | 0.261 | 0.262 | 0.262 | 0.279 | 0.007 |
| *R^2^-overall* | 0.007 | 0.057 | 0.183 | 0.183 | 0.183 | 0.184 | 0.196 | 0.008 |
| Prob. > *F.* | 0.000 | 0.000 | 0.000 | 0.000 | 0.000 | 0.000 | 0.000 | 0.000 |

*Notes*: GLS regressions. Reference group: *Male, Single, Not living with a partner, Live in Urban area, Owned outright,* and *Employed.* Standard errors (clustered at individual level) in parentheses. † *p* < .10; * *p* < .05; ** *p* < .01; *** *p* < .001.

**Table S4. SE cluster at region level and region*wave; DV=GHQ Caseness score**

| *SE cluster level* | Region | | | | Region*Wave | | | |
| --- | --- | --- | --- | --- | --- | --- | --- | --- |
| *Dep. Var.* | Caseness | | Likert | | Caseness | | Likert | |
|  | (1) | (2) | (3) | (4) | (5) | (6) | (7) | (8) |
| Change in duration of time spent at home (%) | 0.025^***^ | 0.024^*^ | 0.035^**^ | 0.042^*^ | 0.025^***^ | 0.024^**^ | 0.035^**^ | 0.042^**^ |
|  | (0.00717) | (0.00989) | (0.0117) | (0.0171) | (0.00677) | (0.00861) | (0.0116) | (0.0143) |
| COVID-19 positive | 0.14^***^ | 0.12^**^ | 0.19^***^ | 0.18^**^ | 0.14^***^ | 0.12^***^ | 0.19^***^ | 0.18^***^ |
|  | (0.0246) | (0.0295) | (0.0455) | (0.0548) | (0.0293) | (0.0329) | (0.0482) | (0.0538) |
| Age | -0.015^***^ |  | -0.033^***^ |  | -0.015^***^ |  | -0.033^***^ |  |
|  | (0.00172) |  | (0.00264) |  | (0.00223) |  | (0.00354) |  |
| Female | 0.55^***^ |  | 0.83^***^ |  | 0.55^***^ |  | 0.83^***^ |  |
|  | (0.0368) |  | (0.0482) |  | (0.0541) |  | (0.0875) |  |
| *Marital status* |  |  |  |  |  |  |  |  |
| Married/civil partnership | -0.16^*^ |  | -0.20 |  | -0.16^*^ |  | -0.20^†^ |  |
|  | (0.0816) |  | (0.131) |  | (0.0629) |  | (0.112) |  |
| Separated/divorced/widowed | 0.0047 |  | 0.066 |  | 0.0047 |  | 0.066 |  |
|  | (0.0792) |  | (0.134) |  | (0.0672) |  | (0.116) |  |
| Living with partner | -0.23^***^ | -0.17^**^ | -0.38^***^ | -0.27^**^ | -0.23^***^ | -0.17^**^ | -0.38^***^ | -0.27^**^ |
|  | (0.0523) | (0.0400) | (0.0666) | (0.0765) | (0.0437) | (0.0517) | (0.0715) | (0.0896) |
| *Education* | -0.29^**^ |  | -0.26 |  | -0.29^**^ |  | -0.26 |  |
| No qualification | (0.106) |  | (0.197) |  | (0.0936) |  | (0.168) |  |
|  | -0.16^*^ |  | -0.18 |  | -0.16^*^ |  | -0.18 |  |
| Other qualification | (0.0678) |  | (0.141) |  | (0.0764) |  | (0.138) |  |
|  | 0.011 |  | 0.051 |  | 0.011 |  | 0.051 |  |
| A level | (0.0621) |  | (0.0967) |  | (0.0640) |  | (0.108) |  |
|  | 0.088^†^ |  | 0.19^*^ |  | 0.088 |  | 0.19 |  |
| Other higher degree | (0.0477) |  | (0.0818) |  | (0.0705) |  | (0.127) |  |
|  | 0.18^**^ |  | 0.17 |  | 0.18^**^ |  | 0.17^†^ |  |
| Degree | (0.0571) |  | (0.104) |  | (0.0573) |  | (0.0988) |  |
|  | -0.025 |  | -0.079 |  | -0.025 |  | -0.079 |  |
| Live in rural area | (0.0507) |  | (0.0923) |  | (0.0442) |  | (0.0744) |  |
|  |  |  |  |  |  |  |  |  |
| *Housing status* | 0.12^*^ |  | 0.20^*^ |  | 0.12^*^ |  | 0.20^*^ |  |
| Mortgage | (0.0541) |  | (0.0868) |  | (0.0544) |  | (0.0979) |  |
|  | 0.34^***^ |  | 0.63^***^ |  | 0.34^***^ |  | 0.63^***^ |  |
| Renting | (0.0698) |  | (0.135) |  | (0.0794) |  | (0.138) |  |
|  |  |  |  |  |  |  |  |  |
| *Employment* | 0.38^***^ | 0.46^***^ | 0.63^***^ | 0.80^***^ | 0.38^***^ | 0.46^***^ | 0.63^***^ | 0.80^***^ |
| Unemployed | (0.0544) | (0.0782) | (0.0982) | (0.140) | (0.0426) | (0.0605) | (0.0782) | (0.115) |
|  | 0.23^***^ | 0.091 | 0.32^**^ | 0.13 | 0.23^***^ | 0.091 | 0.32^***^ | 0.13 |
| Self-employed | (0.0629) | (0.106) | (0.1000) | (0.161) | (0.0515) | (0.0748) | (0.0920) | (0.138) |
|  |  |  |  |  |  |  |  |  |
| *Household composition* |  |  |  |  |  |  |  |  |
| Aged 0-4 | 0.062 |  | 0.070 |  | 0.062 |  | 0.070 |  |
|  | (0.0808) |  | (0.138) |  | (0.0560) |  | (0.0905) |  |
| Aged 5-15 | 0.067^*^ |  | 0.040 |  | 0.067^*^ |  | 0.040 |  |
|  | (0.0281) |  | (0.0397) |  | (0.0322) |  | (0.0501) |  |
| Aged 70 or older | -0.052 |  | -0.063 |  | -0.052 |  | -0.063 |  |
|  | (0.0508) |  | (0.0744) |  | (0.0485) |  | (0.0868) |  |
| Pre-COVID GHQ | 0.38^***^ |  | 0.47^***^ |  | 0.38^***^ |  | 0.47^***^ |  |
|  | (0.00930) |  | (0.00773) |  | (0.0102) |  | (0.00962) |  |
| Long-standing illness or impairment | 0.51^***^ |  | 0.75^***^ |  | 0.51^***^ |  | 0.75^***^ |  |
|  | (0.0677) |  | (0.103) |  | (0.0488) |  | (0.0820) |  |
| Case per 1,000 people | 0.88 | 1.09 | 2.06 | 1.97 | 0.88 | 1.09 | 2.06 | 1.97 |
|  | (1.054) | (1.149) | (2.053) | (2.330) | (0.885) | (0.983) | (1.587) | (1.864) |
| Stringency index | 0.0049 | 0.0056^†^ | 0.0076 | 0.0087 | 0.0049^†^ | 0.0056^*^ | 0.0076 | 0.0087^†^ |
|  | (0.00302) | (0.00294) | (0.00477) | (0.00503) | (0.00280) | (0.00267) | (0.00474) | (0.00475) |
| Constant | 1.22^***^ | 1.57^***^ | 6.45^***^ | 10.4^***^ | 1.22^***^ | 1.57^***^ | 6.45^***^ | 10.4^***^ |
|  | (0.297) | (0.334) | (0.461) | (0.559) | (0.264) | (0.254) | (0.452) | (0.438) |
| Region FE | Yes | Yes | Yes | Yes | Yes | Yes | Yes | Yes |
| Wave FE | Yes | Yes | Yes | Yes | Yes | Yes | Yes | Yes |
| Individual FE | No | Yes | No | Yes | No | Yes | No | Yes |
| Observations | 101236 | 108001 | 101236 | 108001 | 101236 | 108001 | 101236 | 108001 |
| Individuals (cluster) | 12 | 12 | 12 | 12 | 100 | 100 | 100 | 100 |
| *R^2^-within* | 0.019 | 0.019 | 0.013 | 0.013 | 0.019 | 0.019 | 0.013 | 0.013 |
| *R^2^-between* | 0.262 | 0.008 | 0.335 | 0.003 | 0.262 | 0.008 | 0.335 | 0.003 |
| *R^2^-overall* | 0.184 | 0.009 | 0.257 | 0.004 | 0.184 | 0.009 | 0.257 | 0.004 |

*Notes*: GLS regressions. Reference group: *Male, Single, Not living with a partner, Live in Urban area, Owned outright, Employed.* Standard errors in parentheses. † *p* < .10; * *p* < .05; ** *p* < .01; *** *p* < .001

**Table S5. SE cluster at LAD level and LAD*wave; DV=GHQ Caseness score**

| *SE cluster level* | Region | | | | Region*Wave | | | |
| --- | --- | --- | --- | --- | --- | --- | --- | --- |
| *Dep. Var.* | Caseness | | Likert | | Caseness | | Likert | |
|  | (1) | (2) | (3) | (4) | (5) | (6) | (7) | (8) |
| Change in duration of time spent at home (%) | 0.025^**^ | 0.024^*^ | 0.040^**^ | 0.042^*^ | 0.025^**^ | 0.024^**^ | 0.040^**^ | 0.042^**^ |
|  | (0.00905) | (0.00978) | (0.0152) | (0.0165) | (0.00773) | (0.00831) | (0.0127) | (0.0138) |
| COVID-19 positive | 0.14^***^ | 0.12^**^ | 0.19^***^ | 0.18^**^ | 0.14^***^ | 0.12^***^ | 0.19^***^ | 0.18^***^ |
|  | (0.0349) | (0.0415) | (0.0578) | (0.0684) | (0.0314) | (0.0341) | (0.0509) | (0.0550) |
| Age | -0.015^***^ |  | -0.033^***^ |  | -0.015^***^ |  | -0.033^***^ |  |
|  | (0.00214) |  | (0.00378) |  | (0.00202) |  | (0.00360) |  |
| Female | 0.55^***^ |  | 0.83^***^ |  | 0.55^***^ |  | 0.83^***^ |  |
|  | (0.0375) |  | (0.0606) |  | (0.0393) |  | (0.0672) |  |
| *Marital status* |  |  |  |  |  |  |  |  |
| Married/civil partnership | -0.15^*^ |  | -0.18 |  | -0.15^*^ |  | -0.18 |  |
|  | (0.0632) |  | (0.116) |  | (0.0636) |  | (0.111) |  |
| Separated/divorced/widowed | 0.0089 |  | 0.062 |  | 0.0089 |  | 0.062 |  |
|  | (0.0811) |  | (0.141) |  | (0.0777) |  | (0.135) |  |
| Living with partner | -0.23^***^ | -0.17^**^ | -0.38^***^ | -0.27^**^ | -0.23^***^ | -0.17^**^ | -0.38^***^ | -0.27^**^ |
|  | (0.0493) | (0.0629) | (0.0863) | (0.102) | (0.0437) | (0.0530) | (0.0751) | (0.0894) |
| *Education* |  |  |  |  |  |  |  |  |
| No qualification | -0.28^*^ |  | -0.25 |  | -0.28^**^ |  | -0.25 |  |
|  | (0.110) |  | (0.192) |  | (0.101) |  | (0.185) |  |
| Other qualification | -0.17^*^ |  | -0.19 |  | -0.17^*^ |  | -0.19 |  |
|  | (0.0759) |  | (0.137) |  | (0.0789) |  | (0.143) |  |
| A level | 0.012 |  | 0.063 |  | 0.012 |  | 0.063 |  |
|  | (0.0605) |  | (0.106) |  | (0.0621) |  | (0.109) |  |
| Other higher degree | 0.086 |  | 0.20^†^ |  | 0.086 |  | 0.20^†^ |  |
|  | (0.0668) |  | (0.114) |  | (0.0676) |  | (0.120) |  |
| Degree | 0.17^**^ |  | 0.18^†^ |  | 0.17^**^ |  | 0.18^†^ |  |
|  | (0.0583) |  | (0.0966) |  | (0.0559) |  | (0.0971) |  |
| Live in rural area | -0.000032 |  | -0.059 |  | -0.000032 |  | -0.059 |  |
|  | (0.0547) |  | (0.0929) |  | (0.0563) |  | (0.0987) |  |
| *Housing status* |  |  |  |  |  |  |  |  |
| Mortgage | 0.12^†^ |  | 0.20^†^ |  | 0.12^*^ |  | 0.20^*^ |  |
|  | (0.0607) |  | (0.106) |  | (0.0530) |  | (0.0923) |  |
| Renting | 0.32^***^ |  | 0.62^***^ |  | 0.32^***^ |  | 0.62^***^ |  |
|  | (0.0724) |  | (0.132) |  | (0.0687) |  | (0.121) |  |
| *Employment* |  |  |  |  |  |  |  |  |
| Unemployed | 0.39^***^ | 0.46^***^ | 0.64^***^ | 0.80^***^ | 0.39^***^ | 0.46^***^ | 0.64^***^ | 0.80^***^ |
|  | (0.0448) | (0.0748) | (0.0835) | (0.135) | (0.0419) | (0.0610) | (0.0738) | (0.108) |
| Self-employed | 0.22^***^ | 0.091 | 0.30^**^ | 0.13 | 0.22^***^ | 0.091 | 0.30^**^ | 0.13 |
|  | (0.0553) | (0.0982) | (0.0965) | (0.173) | (0.0562) | (0.0855) | (0.0982) | (0.146) |
| *Household composition* |  |  |  |  |  |  |  |  |
| Aged 0-4 | 0.059 |  | 0.065 |  | 0.059 |  | 0.065 |  |
|  | (0.0544) |  | (0.0904) |  | (0.0509) |  | (0.0848) |  |
| Aged 5-15 | 0.066^*^ |  | 0.040 |  | 0.066^*^ |  | 0.040 |  |
|  | (0.0286) |  | (0.0485) |  | (0.0292) |  | (0.0504) |  |
| Aged 70 or older | -0.050 |  | -0.061 |  | -0.050 |  | -0.061 |  |
|  | (0.0445) |  | (0.0764) |  | (0.0417) |  | (0.0697) |  |
| Pre-COVID GHQ | 0.38^***^ |  | 0.47^***^ |  | 0.38^***^ |  | 0.47^***^ |  |
|  | (0.00854) |  | (0.00838) |  | (0.00857) |  | (0.00848) |  |
| Long-standing illness or impairment | 0.50^***^ |  | 0.73^***^ |  | 0.50^***^ |  | 0.73^***^ |  |
|  | (0.0430) |  | (0.0747) |  | (0.0446) |  | (0.0776) |  |
| Case per 1,000 people | 0.71 | 1.09 | 1.46 | 1.97 | 0.71 | 1.09 | 1.46 | 1.97 |
|  | (0.847) | (1.000) | (1.429) | (1.650) | (0.779) | (0.886) | (1.425) | (1.666) |
| Stringency index | 0.0050^†^ | 0.0056^†^ | 0.0077^†^ | 0.0087^†^ | 0.0050^*^ | 0.0056^*^ | 0.0077^*^ | 0.0087^*^ |
|  | (0.00260) | (0.00288) | (0.00425) | (0.00477) | (0.00237) | (0.00248) | (0.00376) | (0.00403) |
| Constant | 0.99^***^ | 1.57^***^ | 5.55^***^ | 10.4^***^ | 0.99^*^ | 1.57^***^ | 5.55^***^ | 10.4^***^ |
|  | (0.297) | (0.311) | (0.498) | (0.524) | (0.476) | (0.256) | (1.055) | (0.424) |
| LAD FE | Yes | Yes | Yes | Yes | Yes | Yes | Yes | Yes |
| Wave FE | Yes | Yes | Yes | Yes | Yes | Yes | Yes | Yes |
| Individual FE | No | Yes | No | Yes | No | Yes | No | Yes |
| Observations | 101236 | 108001 | 101236 | 108001 | 101236 | 108001 | 101236 | 108001 |
| Individuals (cluster) | 369 | 369 | 369 | 369 | 3108 | 3108 | 3108 | 3108 |
| *R^2^-within* | 0.019 | 0.019 | 0.013 | 0.013 | 0.019 | 0.019 | 0.013 | 0.013 |
| *R^2^-between* | 0.279 | 0.008 | 0.350 | 0.003 | 0.279 | 0.008 | 0.350 | 0.003 |
| *R^2^-overall* | 0.196 | 0.009 | 0.267 | 0.004 | 0.196 | 0.009 | 0.267 | 0.004 |

*Notes*: GLS regressions. Reference group: *Male, Single, Not living with a partner, Live in Urban area, Owned outright, Employed.* Standard errors in parentheses. † *p* < .10; * *p* < .05; ** *p* < .01; *** *p* < .001
